# HIV-1 transcription and latency: an update

**DOI:** 10.1186/1742-4690-10-67

**Published:** 2013-06-26

**Authors:** Carine Van Lint, Sophie Bouchat, Alessandro Marcello

**Affiliations:** 1 Université Libre de Bruxelles (ULB), Service of Molecular Virology, Institute of Molecular Biology and Medicine, 12, Rue des Profs Jeener et Brachet, 6041, Gosselies, Belgium; 2 The Laboratory of Molecular Virology, International Centre for Genetic Engineering and Biotechnology (ICGEB), 34149, Trieste, Italy

**Keywords:** HIV-1, Transcription, Post-integration latency, Persistence, Chromatin, Pharmacological strategies, Reservoirs, Therapy, Cure

## Abstract

Combination antiretroviral therapy, despite being potent and life-prolonging, is not curative and does not eradicate HIV-1 infection since interruption of treatment inevitably results in a rapid rebound of viremia. Reactivation of latently infected cells harboring transcriptionally silent but replication-competent proviruses is a potential source of persistent residual viremia in cART-treated patients. Although multiple reservoirs may exist, the persistence of resting CD4+ T cells carrying a latent infection represents a major barrier to eradication. In this review, we will discuss the latest reports on the molecular mechanisms that may regulate HIV-1 latency at the transcriptional level, including transcriptional interference, the role of cellular factors, chromatin organization and epigenetic modifications, the viral Tat *trans*-activator and its cellular cofactors. Since latency mechanisms may also operate at the post-transcriptional level, we will consider inhibition of nuclear RNA export and inhibition of translation by microRNAs as potential barriers to HIV-1 gene expression. Finally, we will review the therapeutic approaches and clinical studies aimed at achieving either a sterilizing cure or a functional cure of HIV-1 infection, with a special emphasis on the most recent pharmacological strategies to reactivate the latent viruses and decrease the pool of viral reservoirs.

## Review

Human Immunodeficiency Virus type 1 (HIV-1) is the cause of the acquired immunodeficiency syndrome (AIDS) and the responsible of a devastating pandemic that affects around 34 million people worldwide (UNAIDS, 2011). Thirty years after the discovery of HIV-1, the virus can still not be cured. Combination antiretroviral therapy (cART) has significantly reduced AIDS-related morbidity and mortality. New regimens are more potent, have fewer side effects and a low pill burden. However, these antiretroviral drugs do not fully restore health or a normal immune status in HIV-1 infected individuals. Patients experience co-morbidities, such as increased cardiovascular disease, bone disorders and cognitive impairment. Interruption of cART almost invariably leads to the re-emergence of detectable viral replication and the progression of AIDS. Moreover, a significant proportion of patients fail to maintain undetectable plasma viral load because of adherence and/or because of the development of drug resistance (reviewed in [1]). Even with optimal treatment and adherence, some patients have problems to keep the virus under control and/or show progressive immune pathology manifesting increased mortality compared to HIV-1 uninfected individuals. This increased mortality is closely associated with inflammation, which persists in cART-treated HIV-infected individuals despite levels of plasma viremia below detection limits. Chronic, pathological immune activation is a key factor in progression to AIDS in untreated HIV-infected individuals. Today, only a small percentage of the HIV-infected people who need treatment worldwide have access to cART. Clearly, more innovative approaches are urgently needed to address these issues.

**Figure 1:**
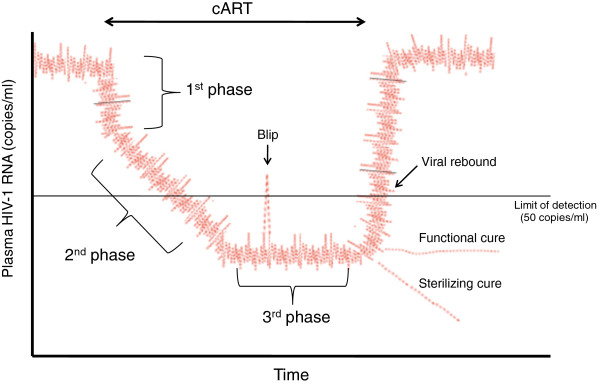
**Dynamics of plasma virus levels in a cART-treated HIV + individuals.** After initiation of cART, viremia undergoes three phases reflecting the decay rates of different populations of HIV-1 latently infected cells. The first phase represents the rapid decay of productively infected CD4+ T cells (activated CD4+ T cells having a half-life of ~1 day). The cells responsible for the second phase, which have a half-life of about 14 days, are not definitively identified (possibly partially activated CD4+ T cells or other cell types such as macropages or dendritic cells). The third phase is a constant phase in which viremia reaches levels below the limit of detection of clinical assays (50 copies viral RNA per ml of plasma). During this plateau phase, occasional viremic episodes (called blips) are detected despite cART. Reservoirs of HIV-1 are responsible for the low but stable level of residual viremia observed during the third phase. This residual viremia is partly derived from the activation of latently infected resting (memory) CD4+ T cells (or subsets of these cells) and partly from another unknown cell source (such as long-lived HIV-infected cells). A rapid rebound of viremia is observed if cART therapy is stopped. Therapeutic strategies achieving control of viremia below detection level after cART cessation could lead to a functional cure. Strategies achieving elimination of HIV-1 from the human body could lead to a sterilizing cure.

Untreated HIV-1 infection is characterized by continuous viral replication that drives CD4+ T cell loss and predicts disease progression. During cART, the plasma virus levels fall below the level of detection of current classical assays (50 copies of viral/HIV-1 RNA per ml of plasma). This decrease goes through several phases of decay corresponding to the half-lives of different populations of HIV-infected cells, which are progressively eliminated (Figure 1). The first decay phase is rapid, being related to the virus produced by activated short-lived CD4+ T cells with a half-life of less than a day in the productively infected state [2]. The second phase reflects virus production by another population of infected cells with a half-life of 1–4 weeks. The third phase is a constant phase with no appreciable decline, caused at least partially by the activation of resting memory CD4+ T cells that start to produce virions. During this constant phase, occasional viremic episodes (called blips) are detected despite prolonged cART treatment from latently infected T cells that became productive after a transient activation of the immune system (i.e. caused by a viral or bacterial infection). Moreover, during this constant phase, a persistent residual low-level viremia (ranging from 1 to 5 copies of viral RNA/ml) can be detected in most patients using ultrasensitive RT-PCR assays. Persistent viremia in cART-treated infected individuals could arise from latently infected cellular reservoirs and/or residual ongoing viral replication.

Two general forms of viral latency (reversibly non productive state of infection) have been observed and can be segregated based on whether or not the virus has integrated into the host cell genome: pre-integration and post-integration latency (reviewed in [3-5]). Pre-integration latency results from partial or complete block of the viral life cycle at steps prior to the integration of the virus into the host genome (incomplete reverse transcription, impaired import of the pre-integration complex into the nucleus or incomplete integration) [6,7]. Unintegrated forms persist in the cytoplasm of CD4+ T cells for only one day and cannot account for the formation of long-term latently infected reservoirs (reviewed in [8]). Although macrophages and some tissues like the brain may retain these forms for a longer period, pre-integration latency appears to be less clinically relevant [9,10]. Indeed, episomal HIV-1 cDNAs are used as surrogate markers for recently infected cells *in vivo*[11,12]. Post-integration latency is a rare event that occurs when a provirus fails to effectively express its genome and is reversibly silenced after integration into the host cell genome. This latent state is exceptionally stable and is limited only by the lifespan of the infected cell and its progeny. Post-integration latency is a multifactorial phenomenon. Mechanisms that maintain HIV-1 latency *in vivo* are incompletely understood. Latently infected cells may be maintained by mechanisms operating at the post-transcriptional level (i.e. inhibition of nuclear RNA export and inhibition of HIV-1 translation by microRNAs). However, in the majority of latently infected cells, HIV-1 infection appears to be blocked at the transcriptional level. HIV-1 transcriptional repression is crucial to the establishment and maintenance of post-integration latency. Several elements contribute to the transcriptional silencing of integrated HIV-1 proviruses (reviewed in [3,13,14]: 1) the site of integration into the host cell genome, the cellular chromatin environment at this site and mechanisms of transcriptional interference; 2) the spatial sub-nuclear positioning of the integrated provirus (reviewed in: [15]); 3) the absence of crucial inducible host transcription factors, such as NF-kappaB (Nuclear Factor Kappa-light-chain-enhancer of activated B cells) or NFAT (Nuclear Factor of Activated T-cells), that are excluded from the nuclei of resting cells and transiently activated by various stimuli; 4) the presence of transcriptional repressors, such as CTIP2 (COUP-TF Interacting Protein 2), DSIF (DRB-Sensitivity Inducing Factor), NELF (Negative Elongation Factor) and the family of TRIM proteins (tripartite motif); 5) the chromatin structure of the HIV-1 promoter and the presence of a repressive nucleosme (nuc-1); 6) the epigenetic control of the HIV-1 promoter (histone posttranslational modifications, such as acetylation and methylation, and DNA methylation); 7) The sequestration of the cellular positive transcription elongation factor b (P-TEFb), composed of cyclin-dependent kinase 9 (cdk9) and human cyclin T1, in an inactive form by the HEXIM-1 (hexamethylene bisacetamide (HMBA)-induced protein 1)/7SK snRNA (7SK small nuclear RNA) regulatory complex; 8) the sub-optimal concentration of the viral transactivator Tat, which promotes transcription by mediating the recruitment to the HIV-1 promoter of the kinase complex P-TEFb, of histone-modifying enzymes and of ATP-dependent chromatin-remodeling complexes required for nucleosomal disruption and transcriptional processivity.

**Figure 2:**
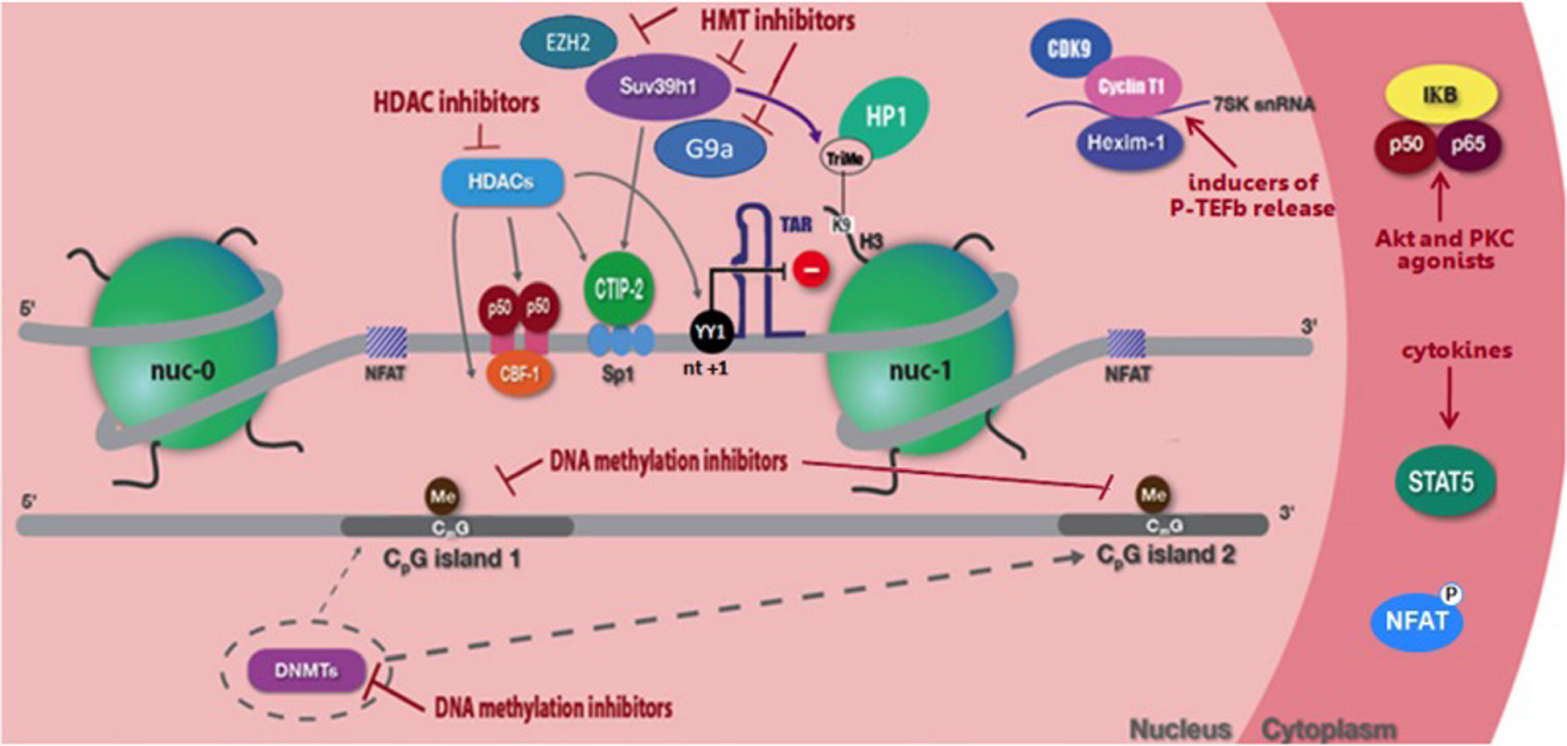
**Reactivation of HIV-1 transcriptional latency.** During latency, nuc-1 blocks transcriptional initiation and/or elongation, Tat is absent and only short mRNAs corresponding to TAR are produced. Nuc-1 is maintained hypoacethylated by HDACs recruited to the 5’LTR via several transcription factor (YY1, CTIP-2, p50-p50 homodimer, CBF-1). The corepressor CTIP-2 interacts with the Sp1 transcription factor at three sites in the HIV-1 5’ LTR and recruits HDACs and the HMT Suv39h1, which trimethylates H3K9 leading to the recruitment of HP1. Other histone methylation repressive marks such as H3K9Me2 or H3K27Me3 catalyzed by the HMT G9a and EZH2, respectively, are also implicated in HIV-1 latency. In addition, during latency, the HIV-1 promoter is hypermethylated at two CpG islands surrounding the HIV-1 transcriptional start site. The dotted arrows indicate that DNMTs are most likely recruited to the HIV-1 promoter but this recruitment has not been demonstrated so far. In latent conditions, the active form of NF-kappaB (p50-p65 heterodimers) is sequestered in the cytoplasm by the inhibitor of nuclear factor kappaB (IκB), while NF-kappaB p50-p50 homodimers occupies the kappaB sites at the viral LTR region. The kappaB sites can also be occupied by CBF-1 and by STAT5Δ/p50 heterodimer in monocytic cells. The phosphorylated form of NFAT is also in the cytoplasm in latency conditions. Moreover, in resting CD4+ T cells, P-TEFb, composed of CDK9 and human cyclin T1, is sequestered in an inactive form by the HEXIM-1/7SK snRNA regulatory complex. In this context, several compounds have been proposed for trasncriptionalreactiation of HIV-1 including HDACIs (SAHA, VPA) to target the hypoacetylated state of nuc-1, HMTIs (chaetocin, BIX-01294, DZNEP) to target HMTs, DNMTIs (5-Aza-CdR) to target 5’LTR DNA methylation, PKC or Akt agonists (sprostratin, bryostatin) to activate the NF-kappaB signaling pathway, cytokines (IL-2, IL-7, GM-CSF) to activate STAT5 and inducers of P-TEFb release (HMBA, JQ1).

Several therapeutic approaches aimed at achieving either a sterilizing cure (in which all replication-competent virus is eradicated, Figure 1) or a functional cure (lack of detectable viremia in the absence of cART despite the presence of replication-competent HIV-1 for prolonged periods together with normal or near normal immunological functions, Figure 1) are under scrutiny (Figure 2 and Table 1). In this context, further understanding of the molecular mechanisms regulating HIV-1 latency (Figure 2) and reactivation from latency in different target cells harboring the virus will help to devise novel strategies to eliminate latent HIV-1 infection or to restrict the latent pool to a size bearable by the host immune system. This could allow individuals to envisage therapeutic interruptions (“treatment-free windows”) and could lead to decrease of the long-term cART side effects and improvement of quality of life.

**Table 1:** HIV cure clinical trials

**Trial/investigator**	**Intervention**	**ClinicalTrials.gov**	**Status**
Optiprim ANRS 147 (A. Chèret)	3 vs 5 ARV at AHI	NCT01033760	Ongoing
IntensVIH (A. Lafeuillade)	RAL + MRV intensification	NCT00935480	Ongoing
Eramune 01 (C. Katlama)	IL7 + intensification RAL/MVC	NCT01019551	Ongoing
Eramune 02 (R. Murphy)	Vacc + intensification RAL/MVC	NCT09976404	Ongoing
S. Deeks	Disulfiram	NCT01286259	Ongoing
D. Margolis	Vorinostat (SAHA)	NCT01319383	[229], Ongoing
S. Lewin	Vorinostat (SAHA)	NCT01365065	Ongoing
L. Østergaard	Panobinostat	NCT01680094	Ongoing
J. Lalezari	ZFN (CCR5)	NCT01252641	Ongoing
P. Tebas	ZFN (CCR5)	NCT00842634	Ongoing
A. Krishnan	Autologous SC with anti-HIV genes	NCT00569985	Ongoing
F. Maldarelli	INFα 2b	NCT01295515	Ongoing
Gilead	Romidepsin	NA	To be started
S. Moreno	Bryostatin	NA	To be started
H. Hatano	Anti-PDI antibody	NA	To be started
A. Woolfrey	Intervention autologous HIV resistant cells	NA	To be started

In this review, we will focus on the latest developments leaving the previous enormous amount of work and discoveries to further reading, including our previous reviews on the same topic [3,5]. We will start discussing the potential sources of residual viremia in cART-treated patients and the nature of the HIV-1 reservoirs and continue with the establishment of *in vitro* models for latency and the study of latency in the patient cells and in animal models. Next, we will describe the recent progress in the molecular understanding of HIV-1 persistence with a special attention to strategies that are being proposed to target these pathways. Finally, we will discuss the therapeutic options that are currently being proposed.

## Potential sources of residual viremia in cART-treated individuals

The low-level residual viremia in cART-treated patients is indicative of active virus production, which can occur without new rounds of infection and propagation of the infection to additional cells. In principle, residual viremia could result from a low degree of ongoing cycles of viral replication (either in the presence of antiretroviral drugs or in anatomical sanctuaries where drug penetration is suboptimal), and/or reactivation of viral expression from latently infected resting CD4+ T cells (harboring stably integrated, transcriptionally silent but replication-competent proviruses), and/or the release of virus from other stable reservoirs.

Concerning ongoing viral replication, it has been demonstrated that productively infected CD4+ T cells persist in the blood and gut-associated lymphoid tissue (GALT) of infected individuals receiving cART. The precise mechanism of this persistence has not been fully delineated but homeostatic proliferation of latently infected resting CD4+ T cells [12] and a sub-optimal intracellular penetration of antiretroviral drugs in lymphoid tissues may contribute to this phenomenon. If viral replication continues during suppressive therapy, low-level viremia would be reduced by the addition of another antiretroviral compound. In this regard, two studies have shown that intensification of conventional cART leads to reduction of the level of cell-associated HIV-1 RNA in CD4+ T cells in the terminal ileum [16] and to the accumulation of circularized 2-LTRs DNA circles in CD4+ T cells in the peripheral blood of some (30%) infected individuals receiving cART [17]. Moreover, the group of David Baltimore has reported that cell-to-cell spread of HIV-1 permits ongoing replication despite cART [18]. However, other intensification studies using Raltegravir have not shown any noticeable diminution of plasma viremia in infected individuals who had been on cART and had maintained <50 copies/ml of HIV-1 RNA ([19-21], abstract 51, session 11 presented at the CROI, 2011). Moreover, most viral evolution studies failed to detect evolutionary genetic changes in the persistent virus population in the majority of cART patients [22-25].

In addition to ongoing viral replication, the cellular latent reservoirs harboring transcriptionally silent but replication-competent stably integrated HIV-1 proviruses are insensitive to cART and able to escape from the host immune response. They are therefore a permanent source for virus reactivation and could be responsible for the rebound of plasma viral load observed after cART interruption.

Altogether, these results indicate that low-level viremia might arise from several different sources including: 1) long-lived HIV-1 infected cells that produce virus; 2) ongoing replication cycles in cells located in sanctuary sites where drug levels are suboptimal such as a tissue-based foci of viral replication within CD4+ T cells and/or myeloid cells; and/or 3) proliferation of latently infected cells with regeneration of a stable reservoir of slowly dividing infected cells.

## Nature of cellular reservoirs

A viral reservoir can be defined as a cell type or anatomical site where a replication competent form of the virus persists for a longer time than in the main pool of actively replicating virus. Over the years, researchers have found that latency can exist in a range of anatomical sites and cell types. The nature of the HIV-1 reservoirs has been recently reviewed [26,27] and will be only briefly summarized here. The most prominent ones are the CD4+ T-cell subsets, primarily resting central memory T cells (TCM) (defined as CD45RA- CCR7+ CD27+) and translational memory T cells (TTM) (defined as CD45RA- CCR7- CD27+) [12,28,29]. A critical issue is where these cells are derived from in the infected patient. Direct infection of quiescent T cells can occur *ex vivo* when cells are treated with a selected set of chemokines [30-33] or by spinoculation [34,35] (see chapter 5). Normally, however, because of the presence of various blocks to the viral lifecycle, HIV-1 is not able to infect quiescent T cells efficiently [36-39]. Hence, latently infected resting memory T cells may be generated when HIV-1 infected actively replicating antigen-stimulated cells survive long enough to revert back to a resting memory state and differentiate into long-lived (half-life of ~44 months) resting memory T cells [40-42]. Unfortunately, to date there is no experimental evidence *in vivo* that can tell us if the latent cells have been directly infected or reversed to the quiescent state with the integrated provirus. The issue is complicated by the fact that the frequency of infected resting memory CD4+ cells is very low *in vivo.* The frequency of latently infected cells, expressed in terms of infectious units per million resting CD4+ T cells, is determined using Poisson statistics and is on the order of 0.1-10 infectious units per million resting CD4+ T cells in most patients on long term cART [28,43,44].

Although the existence of a stable latent reservoir in resting CD4+ T cells is clearly established, evidence for additional reservoirs comes from a detailed analysis of residual viremia. The residual viremia in some patients is dominated by oligoclonal populations called predominant plasma clones that are rarely found in circulating CD4+ T cells [22,45-47]. This phenomenon has suggested the existence of a second reservoir for HIV-1 in a cell type capable of proliferating after infection. Tissue macrophages, primary targets of HIV-1 infection, could be the source of these persistent oligoclonal HIV-1 populations. Indeed, cells belonging to the monocyte/macrophage lineage, are one of the major persistent HIV-1 reservoirs. In these cells, viruses are generally not completely silent but maintain a low level of replication. In contrast to T cells, HIV-1 infection is less cytopathic to these cells; it even extends their lifespan and makes them more resistant to apoptosis. To prove that macrophages function as a reservoir, studies of HIV-1 infection of these cells in various tissues will have to be performed in cART-treated patients who have had prolonged suppression of viremia.

Naïve T cells have also been demonstrated to contain latent proviruses [12,48,49]. HIV-1 DNA persists in naïve CD4+ T cells in patients on suppressive cART, although the frequency of infection is approximately 1–2 logs less than in memory CD4+ T cells [12]. The infection of naïve T cells, which are quiescent in nature, could be explained by their transient partial activation, such as under the influence of cytokines or during thymopoiesis, but they eventually return back to the quiescent (G0) phenotype [48-50]. The HIV-1 DNA concentration has been shown to be similar in CD31+ naïve CD4+ T cells (enriched for recent thymic emigrants) and CD31- naïve CD4+ T cells (naïve cells that have undergone homeostatic proliferation) both prior to and following cART [48,51]. The S. Lewin’s group has shown that the absolute number of infected naïve CD4+ T cells (expressed as HIV-1 DNA copies/ml of blood) in fact increases following cART, suggesting that in the setting of cellular proliferation, the reservoir of infected naïve T cells may expand over time [48].

Patients infected with HIV-1 present hematopoietic abnormalities, which are caused by HIV-1 infection of the bone marrow [52]. Hematopoietic progenitor cells (HPC) have been proposed as a reservoir. A recent study has shown that HIV-1 infects multipotent HPCs and that latent HIV-1 infection is established in some of these HPCs [53]. A follow-up study indicated that CD133+ cord-blood-derived cells were susceptible to *in vitro* infection, but only with X4 tropic virus [54]. However, more recent studies of HPCs in patients on cART have used more rigorous purification of CD34+ cells and have not detected HIV-1 DNA by PCR and therefore not confirmed latent infection of HPCs [55,56]. Further studies will therefore be necessary to determine whether HPCs could constitute a reservoir.

## Anatomic sites of HIV-1 persistence

### The central nervous system (CNS)

An important anatomical site for the HIV-1 reservoir is the CNS, where HIV-infected cells are continuously replenished by circulating infected monocytes. These monocytes cross the blood–brain barrier and differentiate into macrophages and microglial cells. Within the CNS, HIV-1 infection is detected principally in perivascular macrophages and microglial cells [57]. Integrated HIV-1 DNA has also been found in astrocytes from the brains of HIV-infected patients [58], and is associated with HIV-associated dementia [59]. However, it may be important to extend these studies, performed in viremic patients, by studies of astrocytes in patients on suppressive cART. The blood–brain barrier, which restricts entry of cytotoxic T cells and does not allow free flow of anti-HIV-1 drugs, reduces the impact of immune responses and cART on CNS-localized HIV-1 viruses [60,61]. The significance of the CNS viral reservoir is still debated, since the overall share of CNS-derived viruses in persistent viremia appears to be negligible [62].

### The gut-associated lymphoid tissues (GALT)

Another important HIV-1 reservoir is in the GALT, where 5–10 times more HIV-1 RNA than in peripheral blood mononuclear cells can be recovered [16,63]. Moreover, the addition of Raltegravir to patients on suppressive cART results in a non significant decrease in unspliced HIV-1 RNA in the ileum, potentially consistent with ongoing replication at this site [21]. Of note, a recent report has described a lack of evolution in the proviral sequences from recto-sigmoid biopsies [23], results that are inconsistent with continuous viral replication in this site.

Lerner et al. [64] have shown that, in three patients who initiated cART during acute infection, there was no phylogenetic relationship between the HIV-1 RNA sequences from the rebound virus and HIV-1 DNA from the gastrointestinal tract tissue, suggesting that the gastrointestinal tract is not the primary source of rebound viremia after cART interruption cessation. However, these data cannot exclude the possibility that a minor population of the gastrointestinal tract contributes to the rebound of viremia.

## Models of latency

To develop strategies towards a cure, the precise molecular mechanisms responsible for HIV-1 latency and reactivation have to be studied in the more valuable models. Unfortunately, the study of latency *in vivo* has been hampered by the scarcity of latently infected cells, by their difficult enrichment due to the lack of a specific viral marker on the surface thus complicating their isolation from non-infected counterparts and by the high background rate of defective integrated proviruses. Consequently, most of the biochemical studies concerning the molecular aspect of HIV-1 latency until very recently were performed using latently infected transformed cell lines. However, the quiescent phenotype of the latently infected CD4+ T cells found *in vivo* is substantially different from the constitutively activated and proliferating nature of infected cell lines. Since these cell line models do not accurately represent the quiescent cellular environment of primary latently infected cells *in vivo,* the development of improved models *ex vivo* is an important goal for HIV-1 research. Resting memory CD4+ T cells, the major reservoir of latent HIV-1, can be subdivided in central memory T cells (TCM: CCR7+; CD27+) and their derivatives after TCR engagement called effector memory T cells (TEM: CCR7-; CD27-) (for a review: [65]). Subsets of TEM are further characterized by the expression of CCR5, IL-12betaR and intracellular IFNgamma (Th1) or CRTH2 and intracellular IL-4 (Th2). TCM and TEM are CD45RO, while naïve cells express CD45RA. Chomont and collaborators have reported that TCM, together with a subset of cells with functional and transcriptional characteristics that are intermediate between those of TCM and TEM called transitional memory T cells (TTM), are the major reservoir of latent HIV-1 [12]. TEM, naïve T, or terminally differentiated T cells, which are equally susceptible to HIV-1 infection, are not involved in long-term latency during cART. Recent years have shown an effort to establish an experimental system of HIV-1 latency that represents a complete recapitulation of the biologic state of the latent cell reservoir *in vivo*. These systems would be very important for the rational design of drugs to target HIV-1 latency. However, there are inherent difficulties for a unified model system given the presence of multiple cell types where the virus can establish latency and the variety of mechanisms that lead to latency and reactivation. In this context, many laboratories have therefore come up with several informative primary cell-based model systems. In addition to *ex vivo* cellular models, in order to study the properties of reservoirs *in vivo*, models in mice and in primates have been developed.

### *In vitro* models

Most studies of HIV-1 latency have been conducted using latently infected transformed cell lines. However, recent progress in the field allowed the generation of models of latently infected primary lymphocytes.

#### Latently infected transformed cell lines

The study of HIV-1 latency in transformed cell has revealed many insights into the mechanisms of HIV-1 latency, despite the fact that establishment of latency in such systems is often linked to mutations in viral genes or to an effect specific to the site of integration, perhaps not uniformly representative of the quiescent nature of resting CD4+ T cells in patients. Indeed, each latently infected cell line possesses a different HIV-1 provirus (full-length or not) and may contain one or more copie(s) of the integrated provirus in different integration sites or epigenetic environments. Firstly, several models have been developed based on proviruses with minimal HIV-1 features of the LTR/Tat axis from models carrying a full-length provirus. For example, the Jurkat E4 cells and Jurkat bearing an integrated HIV-1 mini-virus are T-lymphoid Jurkat cells containing one single copy of the integrated provirus. However, E4 Jurkat cells possess a fragment of the HIV-1 pNL4-3 (containing *tat*, *rev*, *env* and *vpu*) and a short-lived green fluorescent reporter protein (d2EGFP) in place of the *nef* gene [66,67] The Jurkat cells bearing an integrated HIV-1 mini-virus posses an HIV-1 reporter mini-virus, where luciferase is produced from the HIV-1 5’LTR as an in-frame fusion with p24gag [68].

Secondly, other models have been proposed that are based on full-length proviruses including ACH2, the inducible HIV-rtTA variant, J-Lat T cell lines and U1 promonocytic cell lines. The ACH2 T-cell line [69] and the promonocytic U1 cell line [70,71] show minimal constitutive expression of HIV-1 genes, but a marked activation of viral gene expression following treatment with cytokines or mitogens. However, these models present mutations in Tat (U1) [72] or in its RNA target TAR (ACH2) [73], which have been demonstrated to be causative of the latent phenotype of the proviruses integrated in these two cell lines. Ben Berkhout's laboratory has developed stable cell lines containing an HIV-rtTA variant (in which the Tat/TAR axis transcription motifs have been inactivated and replaced by the inducible Tet-ON system [74]). The HIV-rtTA provirus is completely doxycycline-dependent for virus production, it contains the original transcription factor binding sites in the HIV-1 5'LTR, and infected cells have been obtained without selection steps avoiding any bias towards activation markers [75]. However, the implications of the Tat/TAR axis in reactivation cannot be analyzed. More recently, J-Lat cell lines were developed with an HIV-1-based vector containing an intact Tat/TAR axis [76]. These cells whose unique provirus carries the coding sequence for the green fluorescent protein (GFP) replacing the *nef* gene were selected for a lack of GFP expression under basal conditions [76]. J-Lat cells allow for the rapid assessment of HIV-1 transcriptional activity by cytometric detection of GFP epifluorescence. Consequently, the latently infected transformed cell lines are still useful to study and understand the basic mechanisms governing HIV-1 latency. The first models allow principally the study of HIV-1 promoter activity. The second category permits a complete study of the molecular mechanisms of latency with the influence of produced viral proteins on the HIV-1 promoter but also on host genes.

Interestingly, the J. Karn’s lab has recently generated a HIV-1 latently infected microglial cell line named CHME-5 [77]. To this end, the CHME-5 cell line (primary fetal human microglial cells immortalized with the SV40 large T antigen [78]) was infected with vesicular stomatitis virus G (VSVG)-pseudotyped HIVs bearing a fragment of HIV-1pNL4-3, containing *tat, rev, env,* and *vpu*, plus *nef* adjacent to the reporter gene d2EGFP inserted next to *env.* GFP + cells were next selected by FACS, and further cultured and allowed to enter into a latent state for 4 weeks. Latency of the CHME-5 cell line was characterized by reactivating expression and evaluating nuclear translocation of NF-kappaB [77].

#### Latently infected primary cells

Many laboratories have developed new *ex vivo* experimental primary human CD4+ T cell-based model systems to study HIV-1 latency in a more physiological context (reviewed in [79,80]). Several groups have attempted to mimic *in vitro* the infection of activated CD4+ T cells, with HIV-1 or HIV-1 derived vectors, followed by the transition of these infected cells to a resting state to establish latency in memory CD4+ T cells. These cells are difficult to generate and maintain because most CD4+ T cells die soon after activation if not continuously cultured in the presence of specific cytokines. This results in few cells that have transitioned to a quiescent state and contain integrated HIV-1 that can be used for further study. Culturing of CD4+ T cells in the presence of cytokines, such as IL-2 or IL-7, increases their survival and allows time (several weeks) for more cells to transition to a memory state with integrated HIV. However, IL-2 and IL-7 have been implicated in the reactivation of latent HIV-1 [81-83]. Therefore, these cytokines must be used at a concentration that can maintain CD4+ T cells in culture without reactivating latent HIV. Alternatively, activated and HIV-infected CD4+ T cells can be maintained in culture and allowed to transition back to a resting state in the absence of cytokines by utilizing strategies, such as transduction with anti-apoptotic proteins or co-culture with feeder cell lines [84-86]. All these strategies lead to more viable cells that have been infected and returned to a resting state, allowing for HIV-1 latency to be studied in a physiologically relevant setting.

The first model of HIV-1 latency in primary lymphocytes was developed by Sahu et al. [84] and uses a replication-competent virus for the infection. The feeder cell line H80 (a brain tumor-derived cell line) promotes cell survival in the absence of any cytokine and allows the production of long-lived, mostly central memory CD4+ T cells. However, despite being phenotypically similar to resting cells in many respects, a significant fraction of this population continues to express the early activation marker, CD69, suggesting that these cells are not completely resting. In J. Karn’s laboratory, Tyagi et al. [86] developed a model of latency in which cells are infected with VSV-G-pseudotyped, HIV-1 vectors that lack *env* and encode a fluorescent marker in place of *nef*. Interestingly, they use HIV-1 vectors that express a mutated Tat with the single amino-acid change (H13L) that permits a quicker establishment of the latent state. This model leads to a relatively homogeneous population of central memory CD4+ T cells, which are not completely resting since they continue to express significant levels of the late activation marker, CD25 (CD69 expression is not measured). Marini et al. [87] have developed a primary latency model presenting the advantage of using a replication-competent wild type HIV-1. The majority of cells exhibits a central memory phenotype and is negative for CD25 (CD69 expression is not shown) but is larger and more granular than freshly isolated CD4+ T cells, suggesting that cells may not be completely resting. A disadvantage of this model is the requirement for IL-7, which has been shown to reactivate HIV-1 in latently infected cells [83,88]. Bosque and Planelles [89,90] developed a model in which cells are infected with an envelope-deficient HIV-1 virus that is pseudotyped with HIV-1 Env, thus limiting infection to a single round. Isolated CD4+ T cells were activated first with αCD3/αCD28 antibodies in the presence of IL-2 and then were cultured for several days in three different conditions that produced Th1-helper, Th2-helper, and nonpolarized CD4+ T cells [91]. Biochemical analysis indicated that the Th1 and Th2 populations closely resembled both effector memory and central memory CD4+ T cells, *in vivo*, while the nonpolarized population more closely resembled central memory CD4+ T cells. However, activation markers such as CD25 and CD69 were not assessed before reactivation, giving no indication if these cells were resting or active. Finally, Siliciano and collaborators [85,92] increase cell survival in their model by transduction of a lentivector to express BLC2 prior to primary cells activation and expansion by αCD3/αCD28 antibodies and IL-2. Subsequent infection with a HIV-1 vector lacking several genes (including *gag*, *vif*, *vpr*, *vpu* and *env*) and encoding GFP in place of *nef*, an establishment of latency allowed the screening of a small library of random drug-like molecules for anti-latency compounds. This model generates effector memory cells expressing high levels of CD45RO and low levels of CCR7. A small fraction of them still expresses CD69 and CD25.

At variance with the infection of activated primary cells it is possible to infect directly resting (not activated) CD4+ T cells [30,34,35]. However, this is an inefficient process owing to several blocks imposed by the cellular environment of resting T cells (including lack of dNTPs needed for reverse transcription, a lack of ATP needed for nuclear import of the viral DNA, as well as a restrictive cortical barrier). Swiggard et al. [34] used spinoculation to efficiently deliver large quantities of replication competent HIV-1 virions to freshly isolated resting CD4+ T cells (a mixture of naïve, TCM and TEM cells) in the absence of any activating stimuli. Integration occurred resulting in latently infected cells, albeit at a much lower frequency than activated CD4+ T cells. Alternatively, Saleh et al. [30] have first stimulated freshly isolated resting CD4+ T cells (naïve/TCM/TEM cells) from uninfected donor blood with the CCR7 (a lymphoid organ homing receptor) ligands, CCL19 and CCL21. These chemokines induce neither CD69 nor CD25 expression, but do increase the susceptibility of resting CD4+ T cells to infection by a replication-competent HIV-1 virus. Interestingly, chemokine-induced changes in the actin cytoskeleton that are involved in cell migration appear to be sufficient to allow HIV-1 integration independently of cell activation [32]. Recent results also showed that memory T cells are more sensitive to chemotactic stimulation,which greatly facilitated HIV-1 infection [93]. Hence, there might be a pathway of direct infection of resting T cells also *in vivo* during normal chemokine-directed recirculation of CD4+ T cells between blood and tissue. More recently, by using novel reporter viruses, the W. Greene’s group [35] described an improved version of the primary CD4+ T-cell model originally developed in the O’Doherty’s laboratory [34]. The Green’s model allows the study of latency with replication-competent proviruses in all subsets of CD4+ T cells. In addition to the GFP reporter virus that measures the number of cells in which the latent HIV-1 provirus is successfully reactivated, the Green’s lab has created a luciferase-expressing virus that measures overall levels of transcriptional reactivation of latent HIV-1. Importantly, they also generated a novel mCherry-luciferase dual reporter HIV-1 virus to assess simultaneously the number of cells containing reactivated latent provirus in response of a specific inducer (mCherry) and the magnitude of the response within the entire population (luciferase) [35].

In addition to primary CD4+ T cell models, the Collins’s group has generated an *ex vivo* model for establishing latency in progenitor cells derived from human umbilical cord or bone marrow [94]. CD133+ cells were isolated by magnetic separation and infected with pseudotyped HIV-1 ΔEnv encoding a reporter. Three days after infection, actively infected cells expressing the reporter protein were removed and the remaining cells were resuspended in media with an integrase inhibitor to ensure that increases in reporter gene expression would derive solely from the reactivation of the integrated virus. HIV-1 could establish a latent infection in all of the subsets of HPCs that were examined, including an immature population that includes hematopoietic stem cells and multipotent progenitors. These cells could be reactivated by TNFalpha, SAHA, but not HMBA or 5-aza-2'-deoxycytidine. Although this latter model is preliminary, it appears that differences exist in the establishment of latency in HPCs versus memory T cells that should be analyzed in more detail.

After years when the only experimental models for HIV-1 latency were a series of transformed cell lines carrying an integrated latent provirus, we have entered a new phase with a variety of primary cell-based models. Although it is clear that current research on HIV-1 latency requires such models, the choice of the right system remains difficult. A fundamental issue is that we still don’t know how HIV-1 latently infected resting memory T cells are established *in vivo*. Either route of infection, direct or after reversal to the quiescent state are plausible and can be recapitulated *in vitro*. Since each route may impact on the chromatin environment of the integrated virus, it is likely that the results of experiments aimed at understanding the mechanisms of latency and reactivation will be affected. Another non-trivial aspect to consider is the length of the experiment. *Ex vivo* activation of primary cells, their infection and induction of latency is a lengthy process that takes weeks while direct infection is much quicker. In addition to obvious practical reasons, long term cell culture might induce changes in the chromatin environment of the provirus that do not necessarily mimic the hypothetical *in vivo* situation. On the other hand, techniques such as spinoculation, where a great quantity of virus is forced to infect a resting cell, are not physiological as well.

To conclude, while these primary cell-based models have several attractive features including the ability to rapidly establish latency in memory CD4+ T cells, it will be important to test each model side-by-side with the others. Only by carefully comparing results from the different models to results obtained with cells isolated from blood of cART-treated HIV-infected patients will it be possible to identify meaningful *in vitro* models for *in vivo* HIV-1 latency.

### *Ex vivo* cultures of cells from patients

Viral outgrowth using resting CD4+ T cells isolated from cART-treated aviremic HIV-infected patients seems to be the gold-standard tool for screening and evaluating anti-latency drug candidates, although it is a difficult and costly one. In some of these systems, latently infected cells were treated with a drug candidate to induce virus production, and viral progeny was amplified through co-culture with allogenic, activated, CD8+ depleted PBMCs and in presence of IL-2 [95,96]. Importantly, in contrast to such systems, the Van Lint’s group [97,98] performed *ex vivo* cultures of purified patient cells in the absence of both added IL-2 and allogenic stimulation (i.e. co-culture with PBMCs from an uninfected individual) to avoid extensive nonspecific T-cell activation and proliferation, that may result in the amplification of the genomic viral RNA level. Although assays of resting CD4+ T cells obtained from patients are excellent systems for validating the anti-latency activity of drug candidates, a whole-animal system would allow for a more complete evaluation of reservoirs and potential therapeutic interventions.

### *In vivo* models in mice and in nonhuman primates

Limitations of human clinical studies, especially invasive sampling of multiple reservoirs sites, make it imperative to develop analogous and tractable animal models to carefully characterize viral reservoirs and to rigorously test the efficacy of novel approaches and their effects on tissue reservoirs. HIV-1 cannot be studied in normal mice due to limited species tropism of the virus. Through the pioneering efforts of many investigators, humanized mice are now routinely used to rapidly advance HIV-1 research. The major humanization protocols and contributions to HIV-1 research of each models were reviewed in [99]. Briefly, unlike transgenic mice, models of severe combined immune-deficienthumanized mice (SCID-hu, [50,100,101]) are not genetically manipulated to express human proteins involved in different aspects of the HIV-1 life cycle. Rather, SCID-hu mice models are based on transplantation into immune-deficient mice of either human tissues or hematopoietic stem cells. More specifically, the CB17-SCID-hu mice model transplanted with human fetal thymus and liver tissues under the kidney capsule, allowed the study of thymic infection but there was no systemic reconstitution of the immune system and sites of potential infection were therefore limited [50]. Moreover, since most of the cells generated in this model were naïve, memory cell infection was more difficult to study. Newly developed humanization protocols can overcome some of these limitations: both NOD/SCID BLT mice [99] and hu-Rag2−/− γc−/− mice [102] reconstitute better the human immune system as well as mucosal tissues by transplantation of bone marrow, liver and the thymus under the kidney capsule and by transplantation of bone marrow following irradiation, respectively. In both models, resting memory CD4+ T cells constitute the predominant human T cell population. Moreover, it has been recently demonstrated that cART in HIV-1-infected hu-Rag2−/− γc−/− mice recapitulates some aspects of cART in humans [103]. Complete suppression of viremia on cART and viral rebound following cART discontinuation were observed, suggesting the presence of persistent infection in this model [103].

Of note, Murphy et al. have recently reported findings consistent with feline immunodeficiency virus (FIV) latency in peripheral blood CD4+ T cells isolated from chronically infected cats [104]. This could represent a valuable alternative to primates for the study of HIV-1 latency.

The nonhuman primate models are other important models for HIV-1 cure research (reviewed in [105]). Several similarities between HIV-1 and pathogenic SIV (Simian Immunodeficiency virus) infection of macaques exist including chronic immune activation, mucosal immune dysfunction, microbial translocation and high levels of infection of central memory CD4+ T cells. These nonhuman primate models provide real opportunities for several reasons: (i) identity, dose, and route of virus challenge are known (ii) various clinical parameters such time of infection or duration of cART can be controlled (iii) active and persistent reservoirs can be fully characterized (iv) testing of “risky” interventions is possible. Longitudinal collections of blood/tissue, as well as elective necropsy are available for determining virological and immunological parameters. cART therapy has been modeled using a larger number and variety of anti-retrovirals in nonhuman primates [106,107]. Moreover, an animal model for a functional cure has been developed in rhesus macaques using the SIVagm.sab92018 strain [108]. It allows the study of virological and immunological events that may lead to the infection control both in blood and tissues [108]. Remarkably, Shytaj et al. recently reported complete viral suppression of SIVmac replication in rhesus macaques by a potent multidrug combination [109]. This is an important step in developing an animal model for HIV-1 cure research because it parallels the effects of antiretroviral therapy in HIV-infected humans. Without complete suppression, testing of therapeutic strategies to reduce viral reservoirs is confounded by ongoing cycles of viral replication that can replete such reservoirs.

Since none of the current animal models perfectly reproduce HIV-1 infection and cART, it is likely that several different models will be needed to understand virus persistence, latency, reactivation, and eradication.

## Regulation of HIV-1 gene expression and latency: mechanisms and pharmaceutical targeting

Highly relevant to the transcriptional state of HIV-1 provirus is the chromatin environment that surrounds the viral promoter. Therefore, a big effort is being put in the definition of the integration site-selection preferences of HIV-1 and in the chromatin landscape of the integration site. Since most of the studies for HIV-1 latency have been conducted in T cells, we will focus on those keeping in mind that there might be differences in the mechanisms of latency in other infected cells such as monocytes/macrophages and myeloid dendritic cells.

### Integration-site selection and transcriptional interference

Integration into the host genome is a hallmark of retrovirus infection. After reverse transcription in the cytoplasm the pre-integration complex (PIC) travels to the nucleus where the linear viral DNA is pasted into chromatin. HIV-1 integration is catalyzed by the viral enzyme integrase (IN).

IN operates in close association with the cellular cofactor LEDGF/p75 (lens epithelium-derived growth factor). LEDGF/p75 tethers IN to the host cell chromatin determining HIV-1 integration site distribution, protects it from proteolytic degradation and stimulates its enzymatic activity [110]. Recently, another interactor of IN, the hepatoma-derived growth factor related protein 2 (HRP-2), has been demonstrated to partially complement LEDGF/p75 activity in a LEDGF/p75 knockout cell line [111]. However, another study showed residual integration in double LEDGF/p75 and HRP-2 knockouts indicating that IN alone and/or other host factors may still contribute to the integration specificity [112].

HIV-1 integrates into cellular DNA non-randomly. Bushman and colleagues first demonstrated that HIV-1 integrates preferentially into active cellular genes of transformed cell lines [113,114]. This observation was confirmed in infected CD4+ T cells from patients [115,116]. LEDGF/p75 is a major determinant of HIV-1 integration into transcription units [117,118]. Interaction with LEDGF/p75, together with the local remodeling of chromatin through the interaction with INI1 (a subunit of the SWI/SNF chromatin-remodeling complex), appears to be required locally for integration [119]. However, also the topological organization of chromatin within the nucleus may play a role in target site selection [15]. Depletion of nuclear pore proteins such as RanBP2/Nup358 and the karyopherin Transportin-3/TNPO3 resulted in marked alterations in the distribution of HIV-1 integration sites, providing a link between nuclear entry and integration site targeting [120]. In agreement, the sub-nuclear distribution of integrated HIV-1 in latent cells occurs predominantly at the nuclear periphery [121]. We can envisage that PIC engagement of the nuclear pore addresses HIV-1 integration into active genes localized in its proximity. How does the latency phenotype relate with this topological localization remains open.

The paradox of HIV-1 integration in active genes while being transcriptionally silent stimulated the molecular investigation of the phenomenon. Transcriptional interference has been proposed to explain HIV-1 promoter repression when integrated into introns of highly expressed genes. Transcriptional interference refers to the direct negative impact of one gene on another in *cis*. Convergent antisense transcription results inevitably in inhibition while sense transcription may be inhibitory or stimulatory. Peterlin and coworkers demonstrated HIV-1 promoter occlusion and generation of chimeric transcripts with the endogenous gene in sense integrations [122]. At odds, HIV-1 expression could be enhanced rather than repressed in sense orientation integrations of HIV-1 generated by homologous recombination [123]. However, a bias towards integration in the sense orientation with respect to the endogenous gene has been observed by the same group in an *ex vivo* model system of HIV-1 post-integrative latency [124]. This preference was not observed for acutely infected cells suggesting that sense orientation may provide a more repressive environment for viral transcription than antisense convergent orientation. Recently, Jordan and colleagues explored this phenomenon further and identified chromatin reassembly factors recruited after RNA polymerase II (RNAPII) transcription that repress the integrated cryptic HIV-1 promoter [125]. The picture may be more complicated since it has been observed that the HIV-1 genome can insert in a gene that is also repressed by Tat and this could be an advantage for the virus during transcriptional reactivation [126]. In addition, it has also been shown by allele-specific single cell *in situ* hybridization that transcription of the provirus and of the endogenous gene in which it is integrated may coexist at the same time in the same genomic location.

### Role of cellular factors

Molecular regulation of HIV-1 transcription is a multifaceted process dictated in part by the abundance of cellular transcription factors that induce or repress HIV-1 promoter activity and by the viral Tat protein. HIV-1 promoter activity is also tightly linked to the level of activation of its host cell. Mechanisms that maintain HIV-1 latency *in vivo* are incompletely understood. It is widely accepted that the lack of active forms of key cellular transcription factors (NF-kappaB, NFAT, STAT5, Figure 2) is one element involved in repression of initiation and elongation, respectively, of viral transcription in resting CD4+ T cells (reviewed in [3]). The presence of host transcription repressors (i.e. DSIF, YY1, CTIP2, c-myc, CBF-1, p50 homodimers, Figure 2) may also contribute to HIV-1 latency.

The 5'LTR functions as the HIV-1 promoter and contains DNA binding sites for several ubiquitously expressed cellular transcription factors, such as Sp1 and TFIID, and inducible transcription factors, including NF-kappaB, NFAT and AP-1. Of note, the Berkhout’s group has recently found that the ability of HIV-1 to establish a latent infection is controlled by a four-nucleotide AP-1 element just upstream of the NF-kappaB element in the viral promoter [127]. Indeed, deletion of this AP-1 site deprived HIV-1 of the ability to establish a latent HIV-1 infection. Moreover the extension of this site to a 7 nucleotide AP-1 sequence promoted latency establishment, suggesting that this promoter region represents a latency establishment element. Given that these minimal changes in a transcription factor binding site affect latency establishment to such a large extent, their data support the notion that HIV-1 latency is a transcription factor restriction phenomenon. Indeed, HIV-1 transcription is tightly coupled to the cellular activation status because both NF-kappaB and NFAT are sequestered in the cytoplasm of quiescent T cells and recruited to the nucleus following T-cell activation. The protein kinase C (PKC) pathway leading to the activation of NF-kappaB, NFAT and AP-1 is one of the most important pathway in HIV-1 reactivation (reviewed in [3,128]).

In addition to the binding sites for inducible transcription factors located in the HIV-1 promoter, three AP-1 binding sites have been identified in the coding region of the viral genome, more precisely in a region of the *pol* gene called fragment 5103 [129]. Van Lint and Verdin have previously described an important intragenic region in the HIV-1 genome, whose complete functional unit is composed of the 5103 fragment, the DNase I-hypersensitive site HS7 and the 5105 fragment [130-132]. The intragenic AP-1 binding sites are fully responsible for the PMA-dependent enhancer activity of fragment 5103 and recruit *in vivo* the AP-1 family members c-Fos, JunB and JunD [133]. Moreover, infection of T-lymphoid Jurkat and promonocytic U937 cells with wild-type and mutant viruses demonstrate that mutations of the intragenic AP-1 sites individually or in combination alter HIV-1 replication. Importantly, mutations of the three intragenic AP-1 sites lead to a decreased *in vivo* recruitment of RNAPII to the viral promoter, strongly supporting that the deleterious effect of these mutations on viral replication occurs, at least partly, at the transcriptional level [133]. Single-round infections of monocyte-derived macrophages confirm the importance of the intragenic AP-1 sites for HIV-1 infectivity [133].

Many inducers of the NF-kappaB pathway have been considered for purging the latent reservoirs of HIV-1. PKC agonists, including synthetic analogs of diacylglycerol [134], ingenols [135], phorbol-13-monoesters [136], a jatrophanediterpene (named SJ23B) [137], and the two non tumorigenic phorbol esters prostratin [138,139] and DPP (12-deoxyphorbol 13-phenylacetate) [140], have proven capable of inducing HIV-1 transcription in latently infected CD4+ T cells or in PBMCs from cART-treated patients. PKC agonists downregulate the expression of the HIV-1 receptor CD4 and the coreceptors CXCR4 and CCR5 on the host cell surface [135,141,142]. Therefore, these compounds exhibit interesting bipolar properties as potential molecules to purge resting T-cell latent reservoirs: they upregulate the expression of latent proviruses and inhibit the spread of newly synthesized viruses to uninfected cells via downregulation of critical receptors necessary for viral entry [143]. The phorbol ester prostratin activates HIV-1 expression in latently infected lymphoid and myeloid cell lines and in primary cells [138-141,143-145] with minimal effects on the immune system [144] and causes minimal perturbation of cell cycle progression [145]. Like bryostatin 1 and DPP, prostratin as a PKC activator exhibits non-tumor-promoting activity. The non-mitogenic property of prostratin, its remarkable dual role in activating HIV-1 latently infected reservoirs without spreading infection, its relatively nontoxic behavior, and its ability to act on different cell types make this drug an interesting candidate for viral purging. Despite these numerous advantages, the use of prostratin (and DPP) in human clinical trials awaits safety and toxicity studies in a suitable primate model [146,147]. However, preliminary pharmacokinetic studies are encouraging [138]. Furthermore, chemical synthesis of this therapeutically promising natural compound in gram quantities and at low cost was reported [148] opening the access to numerous new analogs. Interestingly, a recent study [149] has shown that nanoparticles loaded with bryostatin target and activate primary human CD4+ T cells and stimulate latent virus production *in vitro* from latently infected J-Lat 8.4 and 10.6 cell lines and *ex vivo* from latently infected cells in a humanized mouse model, SCID-hu. Moreover, these authors suggest that specific targeting of the nanoparticles to CD4+ T cells by incorporating an anti-CD4 antibody could be an attractive perspective. Interestingly, methamphetamine, a potent CNS stimulant used by certain drug abusers, was recently shown to directly increase HIV-1 LTR activity *in vitro* in human microglial cells (the primary host cells for HIV-1 in the CNS), through an NF-kappaB-dependent mechanism [77]. This finding could explain the more severe HIV-associated neurodegeneration in HIV-infected individuals who abuse methamphetamine than in HIV-infected individuals who do not abuse drugs [150,151]. Because NF-kappaB is a “master regulator” of so many key responses in mammals, inducers of the NF-kappaB pathway for purging the latent reservoir will probably cause side effects. Therefore, a strong but short burst of NF-kappaB activity could be less deleterious until NF-kappaB activation is sufficient to trigger an initial Tat production, which will then fully induce HIV-1 expression. In this regard, a study has demonstrated that a protein secreted by the bacterium *Massilia timonae* efficiently reactivates latent HIV-1 by producing a strong and short activation of NF-kappaB [152]. Other members of the NF-kappaB pathway can also be targeted for HIV-1 transcriptional activation. One of such examples is the IkappaBepsilon protein [153]. Five IkappaB proteins have been described in humans that regulate NF-kappaB signaling. IkappaBs bind NF-kappaB dimers in the cytoplasm, preventing the NF-kappaB proteins from translocating to the nucleus to regulate gene expression. Some NF-kappaB dimers exhibit binding preferences for certain IkappaBs [154]. Thus, the relative abundance of certain IkappaB proteins within the cell may affect the availability of specific NF-kappaB dimers for activation. Moreover, different cell types produce different complements of NF-kappaB dimers [155]. IkappaBepsilon is expressed predominantly in T cells of the thymus, spleen, and lymph nodes [156]. Therefore, major sites and cell types of IkappaB epsilon expression coincide with some of the main reservoirs of HIV-1 latently infected cells. These results may offer attractive therapeutic advantages for HIV-1 activation because IkappaB epsilon is not essential for mammalian growth and development.

The group of A. Jordan has recently identified eight molecules that reactivated latent HIV-1 by screening a library of small molecules. One of them, 8-methoxy-6-methylquinolin-4-ol (MMQO) reactivates HIV-1 in latently infected J-Lat cell lines and in PBMCs from aviremic cART-treated HIV-1 patients without causing T-cell activation and with low toxicity. Interestingly, MMQO produces Jun N-terminal protein kinase (JNK) activation and enhances the TCR/CD3 stimulation of HIV-1 reactivation from latency, but inhibits CD3-induced IL-2 and TNF-alpha gene transcription. Moreover, MMQO prevents TCR-induced cell cycle progression and proliferation in primary T cells [157].

In addition to inducers of HIV-1 gene expression, aloisine A or roscovitine have been shown to inhibit HIV-1 replication when added after HIV-1 reactivation, but inhibition by these compounds is not potent [158]. However, an inhibitor of JNK, called AS601245, has recently been shown to strongly inhibit HIV-1 reactivation by inhibiting AP-1 activation despite high levels of induced NF-kappaB activation [158].

In addition to the PKC pathway, a recent study has shown that depletion of the transcription factors YY1 significantly increases mRNA and protein expression from the HIV-1 promoter [159]. Inhibition of the repressive transcriptional factor YY1 could also be a promising strategy in order to reactivate HIV-1.

Xing et al. have first demonstrated, in a Bcl-2-transduced primary CD4+ T cell model, that Disulfiram (DSF), a FDA-approved drug used to treat chronic alcoholism, reactivates latent HIV-1 without inducing global T-cell activation [160]. Based on this finding, DSF is currently being assessed in a clinical trial for its potential to deplete the latent HIV-1 reservoirs in cART-treated patients (see below). Very recently, the group of J.W. Mellors and N. Sluis-Cremer reported the mechanism by which DSF, an inhibitor of acetaldehyde dehydrogenase, reactivates latent viral expression [161]. This latter study has shown that DSF reactivates HIV-1 expression in the latently infected U1 cell line, but not in the J89GFP or ACH2 cell lines. Interestingly, they found that DSF significantly reduces PTEN (Phosphatase and Tensin homolog, a negative regulator of the Akt signaling pathway) protein levels in U1 cells and in resting CD4+ T cells from HIV-negative donors, resulting in increased phosphorylation of Akt and activation of the Akt signaling pathway [161]. Pharmacological inhibitors of Akt or of NF-kappaB (a downstream target of Akt) block the latent HIV-1 reactivating activity of DSF. In contrast, inhibitors of NFAT, of PKC and of JNK do not affect DSF activity. Neither the J89GFP nor ACH2 cells express PTEN, explaining the lack of DSF effect on HIV-1 expression in these two cell lines [161]. In conclusion, DSF could reactivate latent HIV-1 expression *in vivo* via the Akt signaling pathway through depletion of PTEN and PTEN could be a regulator of HIV-1 latency in cART-treated patients.

T cell factor 4 (TCF-4) is an additional transcriptional factor that inhibits HIV-1 replication [162-165]. TCF-4, a member of the T cell factor/lymphoid enhancer factor (TCF-LEF) family of transcription factors, is a downstream effector of the canonical Wnt/beta-catenin pathway. It associates with beta-catenin (a transcriptional coactivator) to regulate the transcription of a diverse set of genes involved in cell proliferation, differentiation, communication and survival. Binding of a Wnt ligand initiates a cascade of events that results in destabilization of a multiprotein beta-catenin destruction complex and accumulation of a stable, hypophosphorylated beta-catenin that is able to translocate to the nucleus and associate with a member of the TCF/LEF transcription factors family. Within the nucleus, beta-catenin displaces negative regulatory factors from TCF/LEF and recruits cofactors to activate transcription of Wnt target genes. Active Wnt/beta-catenin/TCF-4 signaling pathway plays a significant role in repression of HIV-1 replication in multiple cell targets, including peripheral blood lymphocytes and astrocytes [162,163,166,167]. Recently, Henderson et al. [168] have identified multiple TCF-4 binding sites in the HIV-1 LTR. Deletion or mutation of the site presenting the strongest association, in conjunction with beta-catenin or TCF-4 knockdown in cells stably expressing a LTR reporter construct, enhances LTR basal activity, but has no effect on Tat-mediated transactivation [168]. TCF-4 and beta-catenin at the LTR associate with the nuclear matrix binding protein SMAR1, which likely pulls the HIV-1 DNA segment into the nuclear matrix and away from the transcriptional machinery, leading to repression of basal HIV-1 LTR transcription [168]. Exploring the contribution of beta-catenin/TCF-4 to HIV-1 latency might open novel avenues for anti-latency therapies. In this regard, LiCl, an inhibitor of Wnt signaling, has been recently shown to synergize with histone deacetylase inhibitors (HDACI, valproic acid (VPA) or SAHA) in inducing reactivation of the latent HIV-1 LTR in a cell line model of HIV-1 latency (Rafati et al., 2012, Abstract Retrovirology, 9 (Suppl1):O3)). The level of activation of the latent LTR was found to correlate with the remodeling of the repressive nucleosome nuc-1. Regulators of the Wnt signaling pathway could provide an additional strategy with which to promote reactivation of the latent LTR, particularly in combination with HDACIs.

A member of the Janus kinase (JAK)/signal transducers and activators of transcription (STAT) family, called STAT5, has been demonstrated to play a both positive and negative role in regulating HIV-1 transcription. By using primary human CD4+ T cells transfected with a HIV-1 LTR-driven reporter construct, the full-length STAT5 factor has been reported to upregulate HIV-1 transcription by binding to putative STAT5 consensus binding sites in the LTR U3 enhancer region [169]. STAT5A and STAT5B are activated by a broad spectrum of cytokines such as IL-2, IL-7, IL-15 and granulocyte-macrophage colony-stimulating factor (GM-CSF) through JAK-mediated phosphorylation of a critical tyrosine residue near the C-terminus, leading to the formation of homodimers or heterodimers of STAT5A and STAT5B, followed by nuclear translocation of the active complex [170]. Interestingly, a C-terminally truncated version of STAT5 (STAT5Δ), frequently detected as a dominant isoform in leukocytes of HIV+ individuals [171], has been shown to act as a repressor, rather than an inducer of HIV-1 transcription and expression [172]. Indeed, the Poli’s group has shown that the chronically HIV-1-infected promonocytic U1 cell line mostly expresses STAT5Δ compared to the infected T-lymphoid ACH2 cell line and that GM-CSF stimulation of U1 cells leads to STAT5Δ binding to the LTR U3 region, resulting in a decreased recruitment of RNAPII to the HIV-1 promoter and concomitant repression of viral gene transcription [172]. Moreover, they have very recently reported that, in U1 cells, STAT5Δ interacts exclusively with the NF-kappaB p50 subunit at the level of the nt −85 to −77 HIV-1 subtype B STAT5 site, which overlaps with the nt −81 to -92nt NF-kappaB binding site [173]. Furthermore, GM-CSF stimulation of U1 cells promotes *in vivo* recruitment of p50 to the viral LTR together with STAT5Δ and downregulates HIV-1 transcription. Thus, cytokine-activated STAT5Δ/p50 complexes could contribute to the maintenance of HIV-1 latency in monocytic cells. In addition to STAT5, some other cellular transcriptional repressors of HIV-1 (such as Staf50 [174]) exhibit regulatory function, which is specific for human macrophages [175,176]. Interestingly, IL-7 (a cytokine essential for maintenance of T cell homeostasis) has been shown to induce the expression of latent HIV-1 proviruses in resting CD4+ T cells from HIV-infected patients under cART treatment without global T cell activation, via the JAK/STAT5 signaling pathway [83].

In addition to transcription factors, specific restriction factors exist to defend host cell against retroviral infection. A well-known restriction factor is APOBEC3G that impairs the early phase of the HIV-1 life cycle and may induce latency. Indeed, APOBEC3G strongly inhibits HIV-1 replication in CD4+ T cells by inducing C to U conversions in the viral strand DNA during reverse transcription [177,178]. An inactive form of APOBEC3G can be found in tissue resident naïve or memory CD4+ T cells, which are permissive to HIV-1 infection [179]. In parallel, a growing body of evidence suggests that many members of the TRIM family of proteins constitute an antiviral defense mechanism for the cell [180]. TRIM19 (better known as PML) has been implicated in the restriction of HIV-1 by recruiting Cyclin T1 to nuclear bodies [181]. TRIM22 has also been implicated in the restriction of HIV-1 by repressing the basal LTR activity independently of its E3 ubiquitin ligase activity, of Tat and NF-kappaB-responsive LTR elements [182]. In addition, the group of Cereseto has proposed that TRIM28 inhibits HIV-1 replication by suppressing acetylation of the viral integrase during integration [183]. Moreover, a recent study has reported that ZBRK1 (a KRAB-zinc finger) acts as a transcriptional repressor of HIV-1 LTR in a TRIM28-dependent manner [184]. Indeed, in conjunction with TRIM28 and HDAC2, ZBRK1 suppresses HIV-1 LTR-driven gene expression [184]. Importantly, in elegant studies by the Benkirane’s group and Skowronski’s group, SAMHD1 was recently described as the restriction factor that blocks HIV-1 infection of non-cycling myeloid cells [185,186]. SAMHD1 is a dGTP-dependent deoxynucleotide triphosphohydroxylase [187,188] that reduces the cellular pool of dNTPs in differentiated, non-cycling myeloid cells to levels below those required to support HIV-1 DNA synthesis [188,189]. SAMHD1 also restricts HIV-1 replication in quiescent resting CD4+ T cells by preventing completion of reverse transcription, a finding that could have an important implication in our understanding of HIV-1-mediated CD4+ T-cell depletion and establishment of the viral reservoir [190,191].

Finally, the heat-shock chaperone Hsp90 was shown to bind the HIV-1 promoter and to regulate its expression and viral infectivity [192]. Hyperthermia induces Hsp90 recruitment at the viral LTR, leading to transcriptional reactivation [193]. Since the viral genome exhibits RNAPII pausing at its promoter and NELF-E depletion results in increased viral transcription, it is possible that Hsp90 targets the paused RNA polymerase located on the HIV-1 promoter via NELF and, similar to several genes reported in the work of Paro and collaborators, affects release of paused polymerase from the viral LTR [194].

### Chromatin organization and epigenetic modifications

The chromatin organization and the epigenetic control of the HIV-1 promoter are key elements in viral transcriptional silencing. Previously, the Verdin’s group has shown that two nucleosomes, namely nuc-0 and nuc-1, are precisely positioned in the promoter region of HIV-1 in latently infected cell lines [131,195] and that nuc-1 (located immediately downstream of the transcription start site) imposes a block to transcriptional elongation (Figure 2). Following transcriptional activation, nuc-1 is specifically remodeled [195,196].

Chromatin condensation is critical for the regulation of gene expression since it determines the accessibility of DNA to regulatory transcription factors. Euchromatin corresponds to decondensed genome regions generally associated with actively transcribed genes. By contrast, heterochromatin refers to as highly condensed and transcriptionally inactive regions of the genome [197]. The chromatin condensation status can be modulated through a variety of mechanisms, including posttranslational covalent modifications of histone tails, ATP-dependent chromatin remodeling events and recruitment of repressive factors on methylated DNA [198-200]. ATP-dependent chromatin remodeling complexes couple the hydrolysis of ATP to structural changes of the nucleosome. Histone modifications are all reversible and mainly localized to the amino-terminal histone tails. They include acetylation, methylation, phosphorylation, sumoylation, ADP-ribosylation and ubiquitination. In this review, we will focus on histone acetylation and methylation, the most important histone marks for HIV-1 repression. These covalent modifications of histone tails influence gene expression patterns by two different mechanisms [201]: (1) by directly altering chromatin packaging, electrostatic charge modifications or internucleosomal contacts might emphasize or reduce the access of DNA to transcription factors (such as histone acetylation); (2) by generating interactions with chromatin-associated proteins (such as histone methylation). These modifications function sequentially or act in combination to form the "histone code" and serve as extremely selective recruitment platforms for specific regulatory proteins that drive different biological processes [202]. A description of the different classes of chromatin-modifying enzymes is reviewed in [3]. Here, we describe their importance in HIV-1 repression and their possible therapeutic implications.

#### Histone acetylation

Histone acetyltransferases (HATs) and histone deacetylases (HDACs) influence transcription by selectively acetylating or deacetylating the ϵ-amino group of lysine residues in histone tails. Generally, chromatin acetylation by HATs promotes chromatin opening and is associated with active euchromatin, whereas deacetylation by HDACs diminishes the accessibility of the nucleosomal DNA to transcription factors, thereby generating repressive heterochromatin [203]. Moreover, histone acetylation marks enable the recruitment of bromodomain-containing proteins, such as chromatin remodeling complexes and transcriptions factors, which in turn regulate gene expression. The repressive nucleosome nuc-1 is specifically remodeled following PMA or TNFalpha treatment of the cells, coinciding with activation of HIV-1 gene expression [195]. This remodeling includes posttranslational modifications of histone tails. In this context, Van Lint and Verdin reported for the first time 16 years ago that treatment of latently HIV-1-infected cell lines with two HDAC inhibitors (HDACIs), trapoxin and trichostatin A (TSA), induces viral transcription and the remodeling of the repressive nucleosome nuc-1 [196]. These results indicated that one or several HDACs were bound to the HIV-1 promoter under latency conditions and that deacetylation of the HIV-1 promoter chromatin by these enzymes played a role in the establishment and maintenance of HIV-1 latency. This observation also indicated that treatment of HIV-infected patients with HDACIs might be of therapeutic value by forcing the reactivation of HIV-1 expression and the elimination of the latent HIV-1 reservoir (“flushing hypothesis”) [80,196,204].

Later experiments revealed that transcriptional repressors recruit HDACs to the HIV-1 promoter (reviewed in [3], Figure 2). Following viral reactivation, acetylation of histones and recruitment of HAT at the HIV-1 promoter have been described by the group of Giacca [205]. To date, numerous studies have demonstrated that HDAC1, HDAC2 and HDAC3 are recruited to the HIV-1 LTR5’ and play an important role in viral latency [206-210]. In microglial cells, Marban et al. showed that the corepressor CTIP2 acts as a recruitment platform for HDAC1 and HDAC2 on the HIV-1 promoter, leading to a heterochromatin environment [207,211].

Recently, the group of Poli has shown that essential amino acid restriction results in the transcriptional derepression of silenced transgenes including the HIV-1 provirus in T-lymphocytic cells [212]. This derepression correlates with a significant downregulation of HDAC4. These results indicate that HDAC4 behaves as a critical regulator of exogenous transgene expression [212], sensitive to amino acid starvation and suggest that HDAC4 pharmacological inhibition may be necessary to revert HIV-1 transcriptional silencing and lead to reactivation of latent HIV-1. In this context, recently, Xing et al. have provided strong evidence that HDAC6-selective inhibitor M344 is a potent antagonist of HIV-1 latency, acting by increasing the acetylation of histones H3 and H4 in the nuc-1 region of the HIV LTR [213].

HDACIs present several advantages as a potential inductive adjuvant therapy in association with efficient cART to purge latent reservoirs. They activate a wide range of HIV-1 subtypes [204] without the toxicity associated with mass T-cell activation, which would generate new target cells for neo-synthesized viruses. HDACIs have been demonstrated to repress the coreceptor CXCR4 in a dose-dependent manner [214]. They act on a broad spectrum of cell types; and therefore, in contrast to agents that specifically induce T cells, they could target the different latent reservoirs (macrophages, dendritic cells and other non-T cells). The most important element regarding the therapeutic goal resides in the fact that HDACIs have been safely administrated to patients for several years in other human diseases: phenylbutyrate in beta-chain hemoglobinopathies such as beta-thalassemia and sickle cell anemia [215,216] and VPA in epilepsy and bipolar disorders [217,218]. More recently, SAHA (marketed as Vorinostat) was approved by the Food and Drug Administration (FDA) for treatment of cutaneous T-cell lymphoma [219]. Givinostat and Panobinostat are currently in Phase I/II clinical trials for relapsed hematological malignancies [220,221]. Givinostat has been shown to be more potent than VPA in terms of reactivation of HIV-1 expression *in vitro*[222], while Panobinostat has been shown to be at least 10 times more potent than Vorinostat (reviewed in [223]).

The use of HDACIs as HIV-1 inducers has been well characterized in several latency models and in resting CD4+ T cells from cART-treated HIV-1-infected patients [97,204,222,224,225]. A series of such molecules has been characterized in terms of HIV-1 reactivation [210,226,227]. However, some groups have reported that HDACIs have a lower reactivation potential in a primary latency model [228] or in resting CD4+ T cells from cART-treated HIV-1-infected patients [229] compared to infected transformed cell lines. The first study was performed in a model of primary T cells [228], which might not reflect the *in vivo* situation. Indeed, a recent clinical trial (see below) has shown that Vorinostat reactivates HIV-1 in infected patients [230]. In the second study [229], the concentration of HDACI used was lower than the common concentration used in the numerous studies that have previously tested the HIV-1 reactivation potential of HDACI [226,231]. Moreover, their finding indicates rather that HDACIs induce virus production but from a restricted fraction of infected cells, in agreement with the coexistence of different latency mechanisms [98,99,232].

More recently, many other HDACIs have been evaluated *in vitro* for their reactivation potential in latentlyHIV-1-infected cells: ITF2357 [222], CG05 and CG06 [233], NCH-51 [234] and MC1293 [235]. In order to identify new compounds, several groups have performed high-throughput screening. In this context, molecules such as AV6 [236] or a range of Merck compounds [95] have been highlighted. In parallel, another study has demonstrated that butyric acid naturally produced from bacteria could promote gene expression of latent HIV-1 [237]. The effects of these different compounds have yet to be confirmed in *ex vivo* cultures of resting CD4+ T cells from cART-treated HIV-infected patients.

Latency is a multifactorial phenomenon: different levels of transcriptional and epigenetic blocks are involved and probably act in concert to silence HIV-1 transcription. Attacking simultaneously different levels of latency should be more efficient when viral eradication is the objective since the combination of different types of compounds could synergize in the reactivation of latently infected cells. In this context, Van Lint’s group has reported for the first time that HDACIs and NF-kappaB inducers synergistically reactivate latent HIV-1 [204]. Mechanistically, this synergism was associated with a delayed cytoplasmic reappearance of the inhibitory protein IΚBalpha in response to TNFalpha + TSA versus TNFalpha treatment [238], leading to a prolonged intranuclear presence and DNA-binding activity of NF-kappaB [238,239]. Next, the same group has shown that the non-tumor-promoting NF-kappaB inducer, prostratin, synergistically reactivates HIV-1 production with HDACIs used in human therapies (such as VPA and SAHA) in several postintegration latency model cell lines as well as in *ex vivo* cultures of in CD4+ CD25- CD69- HLADR- resting T cells isolated from blood of cART-treated patients with undetectable viral load [97]. Mechanistically, HDACIs prolonged and increased prostratin-induced NF-kappaB activation and provoked, in combination with prostratin, a more pronounced nucleosome remodeling in the HIV-1 promoter. This study constitutes a proof-of-concept for the coadministration of two different types of therapeutically promising HIV-1 inducers together with efficient cART as a therapeutic perspective to decrease the pool of latent HIV-1 reservoirs [97]. However, in 40% of the cultures, this group could not detect any viral outgrowth following treatment with prostratin and HDACIs individually or in combination. This could result from a stronger epigenetic control of some integrated proviruses. Latter, Burnett and colleagues reported that the same combination prostratin + SAHA synergistically reactivates several HIV-1 subtypes including A, B, C, D and F in primary CD4+ and Jurkat cell-based *in vitro* HIV-1 latency models [240]. Another study has shown similar results with the HDACI MC1293 in a latently infected cell line but did not report testing of this HDACI in *ex vivo* cell cultures from HIV + patients [235]. Interestingly, another non-tumor-promoting PKC inducer, bryostatin, has been shown to activate MAPKs and NF-kappaB pathways and to synergize with HDACIs to reactivate HIV-1 gene expression in latently infected J-Lat cell lines [241].

#### Histone methylation

While histone deacetylation generally contributes to transcriptional repression, histone methylation can be either linked to transcriptional repression or activation, depending on the site of modification. Methylation of H3 at lysine residue 4 (H3K4) is associated with transcriptional activation, while methylation of H3K9, H3K27, and H4K20 is associated with transcriptional repression (reviewed in [242]). Histone methylation marks (including H3K9 dimethylation (H3K9me2), H3K9 trimethylation (H3K9me3) as well as H3K27 trimethylation (H3K27me3)) have been shown to be associated with HIV-1 transcriptional silencing in different postintegration latency models [66,68,207,243,244].

The histone methyltransferases (HMTs) Suv39H1 [243], which is primarily involved in H3K9me3, and G9a [244] which is responsible for H3K9me2, have been demonstrated to play a role in HIV-1 transcriptional silencing (Figure 2). Indeed, Benkirane’s group [243] has shown that Suv39H1, HP1gamma (heterochromatin protein 1) and H3K9me3 mediate HIV-1 repression in different cellular models including PBMCs from HIV+ patients. Moreover, Rohr’s laboratory has previously reported a similar mechanism in *in vitro* HIV-1-infected microglial cells, where Suv39H1 is recruited to the HIV-1 promoter via the transcriptional corepressor CTIP2 leading to a multi-enzymatic complex including HDAC1, HDAC2 and HP1 [207,211]. In this context, a study has very recently reported increased CTIP2, HP1, MeCP2 and HDAC1 levels in postmortem brain tissue from HIV+ latent cases, most probably due to posttransciptional mechanisms [245]. In addition, G9a-mediated H3K9me2 can also recruit HP1 and therefore participate in the maintenance of HIV-1 silencing [244]. Results from Ding et al. suggest that the G9a-like-protein (GLP) may also play a significant role in the maintenance of HIV-1 latency by catalyzing H3K9me2 in new clonal cell lines where the provirus is integrated in active gene regions and presented an hypomethylation status [246]. Together, their data suggest that histone methyltransferase inhibitors (HMTIs) could represent promising therapeutic drugs in strategies aimed at purging the HIV-1 latent reservoirs.

Two specific HMTIs have been described so far: chaetocin and BIX-01294. Chaetocin, a fungal mycotoxin from *Chaetomium minutum*[247], acts as a specific inhibitor of Suv39H1 in a S-adenosylmethionine (SAM)-competitive manner [248]. Chaetocin belongs to 3-6-epi-dithio-diketopiperazines, which have been previously reported to have biologic effects including immunosuppressive [249], anti-inflammatory [250] and/or antiviral [251] activities. BIX-01294, a diazepin-quinazolin-amine derivative, functions as a specific inhibitor of G9a [252] in an uncompetitive manner with SAM by binding the G9a SET catalytic domain [252,253]. Recently, Van Lint’s group demonstrated that the HMTI chaetocin induces HIV-1 recovery in 50% of the CD8+ depleted PBMCs cultures tested and in 86% of the resting CD4+ T-cell cultures from HIV-1+ cART-treated patients with undetectable viral load [98]. They next confirmed the high reactivation potential of HMTIs using BIX-01294, which induced HIV-1 recovery in 80% of the patient resting memory CD4+ T-cell cultures tested [98]. This study and several other recent studies demonstrate that the specific HMTIs chaetocin and BIX-01294 reactivate latent HIV-1 with minimal effects in different latently infected cellular models compared to the effects observed in patient cells [66,68,98,244]. Moreover, the Van Lint’s group has shown that the combinations chaetocin + prostratin and chaetocin + SAHA causes, in most cases, a higher HIV-1 reactivation than these compounds used alone [98], in agreement with the fact that a combination of two different HIV-1 inducers can act on different mechanisms of latency. They also observed this effect using the combination BIX-01294 + SAHA in a smaller group of patient cell cultures. In conclusion, the Van Lint’s group showed for the first time that HMTIs, alone or in combination with HIV-1 inducers, cause HIV-1 recovery in resting memory CD4+ T cells from cART-treated patients [98]. Although chaetocin and BIX-01294 cannot be safely administered to humans, the latter results constitute a proof-of-concept for the use of HMTIs in strategies aimed at reducing the pool of HIV-1 latent reservoirs. Since HMTIs also represent promising compounds in anti-cancer therapies [254-256], other safer HMTIs should be synthesized soon and evaluated for their reactivation potential in cells from HIV-1+ cART-treated individuals.

Suv39H1-mediated trimethylation requires previous demethylation of H3K4 by Lysine-specific demethylase 1 (LSD-1/KDM1) [257]. The Rohr’s and Van lint’s groups have recently postulated that this histone demethylase may be part of the multienzymatic complex recruited by CTIP2 to the HIV-1 promoter [258]. They have reported that LSD1 functionally cooperates with CTIP2 in a synergistic manner to repress both viral replication and transcription in microglial cells, the main HIV-1 targets in the central nervous system [258]. They also demonstrated that CTIP2 and LSD1 interact by coimmunoprecipitation experiments and colocalize in cells with the HIV-1 Tat protein within heterochromatic nuclear structures by confocal microscopy experiments [258]. Altogether, their results support the idea that LSD1 and CTIP2 cooperate to repress the HIV-1 promoter. Recruitment of LSD1 at the HIV-1 proximal promoter is associated with induction of both H3K4 and H3K9 trimethylation in the U1 monocytic/macrophage cell line. Mechanisms underlying LSD-1-mediated increase in H3K4 trimethylation might rely rather on the ability of LSD1 to recruit hSET1 and WDR5, two members of the hCOMPASS complex, at the promoter than on its own enzymatic demethylase activity [258]. These data suggest that the transcriptional repressor LSD1 constitute a potential target (at least in infected cells of the monocytic/macrophages lineage) to induce HIV-1 latency with histone demethylase inhibitors. In contrast, in latently infected T cells, the LSD1/KDM1/CoREST complex, normally known as a transcriptional suppressor, was shown to act as an activator of HIV-1 transcription through specific demethylation of K51 in Tat [259]. The two studies thus observed opposite effect of LSD1 in microglial cells versus in T cells. This could be due to cell type-specific differences that should be further investigated.

In addition to H3K9 methylation, the Karn’s laboratory has elegantly demonstrated that the HMT enhancer of Zeste 2 (EZH2, a HMT that is part of the Polycomb Repressive Complex 2, called PRC2), the enzyme responsible for H3K27me3, is present at high levels at the LTR of silenced HIV-1 proviruses and was rapidly displaced following proviral reactivation in T cells (Figure 2[66]). This correlates with H3K27 trimethylation of the HIV-1 promoter in latency conditions, inducing a repressive chromatin structure, and with a decreased H3K27me3 level in activated conditions [66]. Recently, the group of Tyagi has reported that CBF-1 (C-promoter binding factor-1) is responsible for the recruitment of EZH2 and other chromatin-modifying enzymes of the Polycomb complex to the HIV-1 promoter [27]. The complex including EZH2 is very important because it can serve as a binding platform for multiple histone- and DNA-modifying enzymes [260,261]. Treatment of cells with the broad spectrum HMTI DZNep has shown HIV-1 reactivation of silenced proviruses and a significant inhibition of EZH2 activity in a primary T-cell latency model [66]. However, DZNep is not a specific inhibitor of EZH2 but possesses only a greater inhibitory potency for EZH2 than for other HMTs. Indeed, DZNep is an inhibitor of S-adenosyl-L-homocysteine (SAH) hydrolase and thus inhibits indirectly EZH2 through effects on intracellular SAH concentrations and promotes degradation of the PRC2 complex [262]. EZH2 is also implicated in tumorigenesis and correlates with poor prognosis in several tumor types [263-266]. In this context, a recent study identified specific inhibitors of EZH2 methyltransferase activity by a high throughput biochemical screen [267]. In a linked study, a small molecule of this screen has been optimized resulting in a highly selective, potent, SAM-competitive inhibitor of EZH2, named GSK126 [268]. They demonstrated that GSK126 decreases global H3K27me3 levels and reactivates silenced PRC2 target genes in lymphoma. GSK126 has been also evaluated in mice where it was well tolerated and similar results were observed in cell culture [268]. Therefore, it would be interesting to evaluate the GSK126 molecule for its reactivation potential on latent HIV-1 in infected patient cell cultures.

#### DNA methylation

In mammalian cells, DNA methylation occurs as 5-methyl cytosine predominantly in the context of CpG dinucleotides and is achieved by the specific recruitment of DNA methyltransferases (DNMTs). DNA methylation in transcriptional regulatory regions is generally associated with gene silencing, either by directly blocking binding of transcription factors to their recognition sequences or by indirectly preventing transcription factors from accessing their target sites through attachment of methyl-CpG-binding proteins (MeCPs) that “read” DNA-methylation patterns. These MeCPs recruit HDACs and HMTs, thereby resulting in formation of a closed repressive chromatin structure (for review [269]). During latency, the HIV-1 promoter is hypermethylated at two CpG islands surrounding the HIV-1 transcriptional start site as demonstrated both in J-Lat cell lines and in the Planelles’s primary T cell model of HIV-1 latency ([270], Figure 2). In J-Lat cells, methyl-CpG binding domain protein 2 (MDB2) and HDAC-2 are recruited to the promoter via the second CpG island [270]. Treatment with 5-aza-2’deoxycytidine (5-Aza-CdR, decitabine) decreases cytosine methylation in the two HIV-1 CpG islands, resulting in loss of MBD2 and HDAC-2 from CpG island 2 of the viral promoter region and in partial transcriptional reactivation [270]. The group of Hirsch compared the CpG dinucleotide methylation pattern of the HIV-1 promoter in aviremic versus viremic HIV-1-infected individuals by bisulfite sequencing of DNA from memory resting CD4+ T cells [271]. Analysis of the CpG methylation profile has shown that the HIV-1 5’LTR in 6 patients without detectable plasma viremia contain from 19% to 100% of methylated CpG compared with less than 0.1% for the control group of viremic patients. These results indicate that HIV-1 promoters in the long-term cART-treated aviremic individuals are hypermethylated, in contrast to non-methylated promoters of viremic patients [271]. However, more recently, J. Blazkova now in Fauci and Chun’s laboratory reported novel contrasting results. Indeed, they examined the methylation status of the first CpG island by bisulfite sequencing in resting CD4+ T cells from 11 aviremic patients and did not find significant 5’LTR methylation in any of them [272]. They observed a median frequency of methylated CpG dinucleotides within the HIV-1 5’LTR of 2.4% (range 0-10%) [272]. Both groups used the same technique of DNA methylation analysis and the same system of controls based on Jurkat clones. The major sources of differences between both studies could originate from variability in patients characteristics and cART treatments. The patients from the Hirsh’s study with the most heavily methylated 5’LTRs were long-term infected individuals (infected for 11, 12 and 16 years, with a median of 10 years of ART). Some of them were treated by inefficient therapy at the beginning (see Supplementary Table 3 in [271]). In contrast, the longest treatment period in the Fauci’s study was 6.6 years with a median of 2.9 years. Another difference between both studies is the population of analyzed CD4+ T cells: HIV-1 proviruses analyzed in the Hirsh’s study were extracted from memory CD4+ T cells, while HIV-1 proviruses analyzed in the Fauci’s paper were obtained from resting CD4+ T cells. More stringent mechanisms of maintenance of HIV-1 latency, including CpG methylation, may be needed in the memory CD4+ T cells, which are more prone to proliferate than the resting CD4+ T cells. Since DNA methylation may be a late event that enhances silencing of already-latent viruses rather than contributing to entry into latency, the time of infection in each patient may be an important factor contributing to the differences between both studies. Indeed, substantially longer period of selection of latent proviruses in the memory cell population [271] can result in accumulation of HIV-1 proviruses with methylated promoters in comparison to non-methylated HIV-1 proviruses harbored in the shorter-term-selected population of resting cells [272]. Very recently, Palacios et al. have compared the methylation state of the HIV-1 promoter in PBMCs from two group of infected patients: 1) long-term nonprogressors and/or elite controllers (the LTNP/EC group) who maintain undetectable or low levels of viremia (< 50 or 2000 RNA copies/ml, respectively) without treatment, and 2) aviremic patients in whom cART results in undetectable plasma viremia. Palacios et al. have shown that the 5’LTR of HIV-1 in all aviremic patients exhibited very low or no methylation at all (range of 0 to 1%) compared with that the LTNP/EC group, where the percentage of methylated CpGs in the HIV-1 promoter was higher (median of CpG methylation of 5.4%). However, it is difficult to compare this latter study with the two previous ones [271,272] because bisulfite analysis was performed on total PBMCs (containing other infected cells such as monocytes in addition to the CD4+ T cells) instead of memory CD4+ T cells. Palacio et al. have observed a weak 5’LTR DNA methylation level in the LTNP/EC group, thereby strengthening the hypothesis that DNA methylation is an epigenetic mark associated with certain forms of latency. Evidently further studies will be necessary to clarify the role of DNA methylation in HIV-1 latency *in vivo*.

Various anticancer agents including 5-aza-2'deoxycytidine (5-Aza-CdR), an FDA-approved inhibitor of DNA methylation used in humans to treat myelodysplastic syndrome (marketed as Decitabine) [273], were shown to induce HIV-1 transcription in latently infected cell lines [274] and in a doxycycline-dependent HIV-rtTA variant [76]. However the role of DNA methylation in HIV-1 latency is still controversial, and some laboratories have even reported that CpG methylation did not correlate with transcriptional silencing [275]. In addition to the four well-characterized nucleoside analog methylation inhibitors (i.e. 5-azacytidine (5-Aza), 5-Aza-CdR, 5-fluoro-2'-deoxycytidine, and zebularine), there is a growing list of non-nucleoside DNA methylation inhibitors such as procaine, procainamide, hydralazine and RG108 [276]. Only 5-Aza and 5-Aza-CdR are currently FDA-approved and used in cancer therapies. The major hindrance of their usage in humans is their instability *in vivo* and the toxicity secondary to their excessive incorporation into DNA, which causes cell cycle arrest. These cytosine analogs have also been demonstrated to induce proteasomal degradation of DNMT1 [277]. Today, no clinical trial with HIV-1-infected patients has been performed using DNA methylation inhibitors to reduce the pool of latent reservoirs, but it could an interesting approach.

In addition to their DNA methylation inhibitory activity, 5-Aza and 5-Aza-CdR were reported to exhibit another potential advantage in the context of strategies aimed at curing HIV-1 infection. Indeed, it was reported that the primary antiviral mechanism/activity of 5-Aza (after reduction to 5-Aza-CdR) and of 5-Aza-CdR could be attributed to their ability to increase the HIV-1 mutation rate through viral DNA incorporation during reverse transcription [278]. This results in decreased HIV-1 replication and infectivity through lethal mutagenesis. Clouser et al. have shown that a combination of two clinically approved drugs, decitabine and gemcitabine (2'-deoxy-2',2'-difluorocytidine, a nucleoside analog also presenting ribonucleotide reductase inhibitory activity), reduced HIV-1 infectivity by 73% at concentrations that had minimal antiviral activity when used individually [279]. Recently, the same group has shown that 5-Aza-CdR in combination with gemcitabine, a FDA-approved drug, inhibited disease progression at doses that were not toxic in murine AIDS model, as detected by histopathology, viral loads, and spleen weights [280]. Resveratrol (a ribonucleotide reductase inhibitor) was also shown to inhibit HIV-1 infectivity in combination with 5-Aza-CdR [281].

Combinations of a DNA methylation inhibitor with a HDACI has been previously used in clinical trials as anticancer treatments. A combination 5-Aza-CdR/VPA has been tested in latently infected cell lines but failed to synergistically reactivate HIV-1 transcription [270]. However, in the same experiment, the authors showed that inhibiting provirus methylation led to an almost complete reactivation of latent HIV-1 in J-Lat T cells when combined with the NF-kappaB signaling activator TNFalpha [270]. In another study, 5-Aza-CdR was also shown to synergize with prostratin, which triggers reactivation of latent HIV-1 without broad T-cell activation and inhibits *de novo* virus infection [144]. Fernandez and Zeichner have shown that 5-Aza-CdR plus TNFalpha activates HIV-1 at least twice as well as TNFalpha alone in almost all J-Lat cell lines tested but the J-Lat 10.6 cell line, in which TNFalpha plus 5-Aza-CdR moderately decreases activation compared to the activation observed after treatment with TNFalpha alone [282]. Surprisingly, 5-Aza-CdR decreased TNFalpha-induced activation of HIV-1 gene expression to an even greater extent in the latently infected cell lines ACH2, J1.1 and U1 than in the J-Lat 10.6 cell lines [282]. In certain cell lines, the authors also observed that 5-Aza-CdR decreases induction of viral expression by the HDACI TSA [282]. These differential responses to epigenetic inducers observed in the various cell lines may be due, in addition to the direct effect of the epigenetic drugs on LTR demethylation, to different indirect effects on cellular genes that directly or indirectly inhibit HIV-1 transcription. Recently, the Van Lint’s group has shown that 5-Aza-CdR in combination with the HDACI SAHA synergistically induces HIV-1 gene expression in several J-Lat cell lines (Bouchat and Van Lint, unpublished data). The reactivation potential of 5-Aza-CdR is currently tested in combination with others HIV-1 inducers in *ex vivo* cultures of resting CD4+ T cells from cART-treated HIV-infected patients with undetectable viral load (Bouchat and Van Lint, unpublished data). The use of DNA methylation inhibitors coupled with a PKC agonist or with an HDACI could be a further step to purge the latent reservoirs in cART-treated patients.

### The viral protein Tat and associated cofactors

Tat is the viral trans-activator of transcription that binds the transactivation response RNA element (TAR, Figure 2) at the 5’-end of all viral transcripts to promote transcriptional elongation (for a review: [283]). Tat is a promiscuous viral protein that has been show to associate to a number of host factors. However, the first comprehensive protein-protein interaction network of HIV-1 infected cells showed a remarkably low number of hits for Tat, which included the well described Cyclin T1 and CDK9, but excluded several others [284]. The levels of Tat in an infection are certainly physiologic, may be too low at the time of harvest to be able to pull down all the possible interactors. Indeed, several recent studies identified important Tat cofactors by overexpressing tagged-Tat alone in cells (see below). Furthermore, careful inspection of the crude data of Krogan’s work shows the presence of some already identified factors and the criteria of inclusion for the final network representation should be analyzed carefully. For example, the nucleosome assembly factor 1-like 1 (NAP1L1), which was previously shown to functionally interact with Tat, can be found also in Krogan’s pulldowns associated to Tat and other viral proteins and therefore exclude for specificity reasons [285,286] To note that NAP1L1 was also described as functional interactor of the Rev protein [287]. In the following chapters, we will focus on Tat and its partners according to the latest information available.

#### Tat and P-TEFb

The HIV-1 promoter is a widely used model for mammalian RNAPII elongation control and has provided several insights in the general mechanism of cellular transcription. In the absence of the viral transactivator Tat, basal transcription from the long terminal repeat (LTR) leads to RNAPII pausing after synthesis of a short RNA that includes TAR. The negative elongation factors NELF and DSIF induce RNAPII pausing on the promoter but few molecular details of this process are known (reviewed in [288]). Kiernan and collaborators recently proposed that also RNA contributes to pausing [289]. The recruitment of the Microprocessor complex, Rrp6 exoribonuclease and of the termination factors Setx and Xrn2 induces the cleavage of the short TAR hairpin leading to premature termination by an RNAi independent mechanism. This mechanism is active also on a subset of cellular genes reinforcing the notion that post-initiation events are a hallmark of cellular gene regulation. Indeed, genome-wide mapping studies revealed promoter-proximal pausing of RNAPII on a large proportion of human genes (ENCODE Transcriptome Project, 2009; [290-292]).

Tat, together with P-TEFb, binds the stem loop within TAR, allowing cdk9 to phosphorylate the RNAPII carboxyterminal domain (CTD) and the negative transcription elongation factors NELF and DSIF, licensing RNAPII for productive elongation (Reviewed in [293,294]). Tat recruits Cyclin T1/cdk9 either from the inactive complex containing the 7SK snRNA, HEXIM1 (or his homologue HEXIM2) (Figure 2), the La-related protein 7 (LARP7), and the 7SK-specific methyl-phosphate-capping enzyme (MePCE) or from the Cyclin T1/cdk9 complex containing the bromodomain-containing protein 4 (BRD4) active on cellular genes. However, other Cyclin T1/CDK9-associated protein complexes that negatively modulate Tat activity have been recently identified [295,296]. Frankel’s group reported the unexpected findings that Tat assembles into a complex with P-TEFb in its inactive 7SK snRNP form and that this complex is recruited to the HIV-1 promoter before transcription initiation in a TAR-independent manner. Once transcription begins, the nascent TAR hairpin is synthesized and is required to displace the inhibitory 7SK snRNP complex and activate the P-TEFb kinase, which occurs when Tat and CycT1 bind to TAR [297].

Recent advances in live cell imaging allowed direct measurements of RNA biogenesis from the HIV-1 promoter exceeding 50 kb min^-1^[298]. The studies of Marcello and collaborators demonstrate that Tat-mediated HIV-1 transcription is highly efficient and able to produce a large amount of pre-mRNA in a short time [299]. Tat is such an effective elongation factor possibly because it is able to recruit the “super elongation complex” (SEC) in addition to P-TEFb. Independent work by the Benkirane and Zhou laboratories using an affinity-purification strategy led to the identification of ELL1 and its homolog ELL2, AFF1 and its homolog AFF4, ENL, AF9 and components of the polymerase-associated factor complex (PAFc) as SEC components of the Tat/P-TEFb complex [300,301]. ELL1 and ELL2 are well-characterized transcription elongation factors that stimulate the processivity of RNAPII and act in concert with P-TEFb. The SEC was also independently identified associated to the mixed-lineage leukemia (MLL) protein to promote expression of MLL-dependent genes [302]. However, SEC components ENL/AF9/AFF4 interacting with PAFc/RNAPII are able to promote elongation of genes independently of Tat/MLL and were shown to increase HIV-1 basal transcription [303]. Control of SEC by ubiquitination and its reactivation by prostratrin and HMBA (Hexamethylene bisacetamide) represents a novel avenue for developing strategies to control HIV-1 transcription.

HMBA, a hybrid bipolar compound, is a clinically tolerable agent, which was first developed as an anticancer drug [304,305]. HMBA was previously shown to reactivate viral production in chronically infected cell lines [306,307]. HMBA activated transiently the PI3K/Akt pathway, which leads to the phosphorylation of HEXIM1 and the subsequent release of P-TEFb from its transcriptionally inactive complex with HEXIM1 and 7SK snRNA [308]. Moreover, HMBA triggers Cdk9 recruitment to the HIV-1 5'LTR via an unexpected interaction with the transcription factor Sp1, resulting in stimulation of transcription elongation and viral production [309]. HMBA was shown to induce HIV-1 gene expression in the latently infected T-lymphoid (ACH2, Jurkat cells) and U1 monocytic cell lines and in resting CD4+ T cells isolated from PBMCs which were infected *in vitro* with HIV-1_LAI_[308]. Moreover, Klichko et al. have reported that treatment with HMBA downregulates expression of CD4 in PBMCs, but does not alter expression of the HIV-1 co-receptors CXCR4 and CCR5 [310]. However, unlike mitogen activation, HMBA did not increase cell susceptibility to HIV-1 infection or the expression of cell surface markers of activation [310].

In order to affect the P-TEFb equilibrium, five independent studies have very recently reported that JQ1 (an inhibitor of BET bromodomain BRD4 [311]) is able to reactivate latent HIV-1 [312-316]. The most recent study has also shown that 3 other BRD4 inhibitors (I-Bet, I-Bet151 and MS417) reactivate HIV from latency [316]. The bromodomain containing protein BRD4 and its inhibition of Tat-transactivation is a major impediment to latency reactivation [313]. BRD4 competitively blocks the Tat-SEC interaction on the HIV-1 promoter [313]. The BET bromodomain inhibitor JQ1 dissociates BRD4 from the HIV-1 promoter to allow Tat recruitment of SEC to stimulate HIV-1 elongation. Others studies have shown that JQ1 transiently increases levels of free P-TEFb and BRD4/P-TEFb and SEC/P-TEFb complexes in cells [312]. Indeed, although primarily thought of as an epigenetic modifier, JQ1 also affects the P-TEFb equilibrium directly by removing P-TEFb from the 7SK snRNP or by an indirect effect via stress, as the structure of chromatin changes upon release of BRD4. The BRD4 loss enhances HIV-1 gene expression by increasing Tat/P-TEFb association and RNAPII transcriptional elongation [315]. Importantly, JQ1 was shown to reactivate latent HIV-1 in several cellular models for postintegration latency: in T lymphoid latently infected cell lines [312-315], in the latently infected U1 monocytic cell line [315], in a primary T-cell model of HIV latency [316], and in *ex vivo* cultures of patient cells [314,315]. The Montano’s group has reported HIV-1 recovery in one of the three resting CD4+ T cell *ex vivo* cultures prepared from HIV-1-infected cART-treated patients [314]. The Brass’s group has tested the reactivation potential of JQ1 in *ex vivo* cultures of CD8+ depleted PBMCs isolated from 19 cART-treated patients with undetectable viral load and has shown that JQ1 alone does not induce HIV-1 recovery but potentiates the actions of several known HIV-1 inducers (PHA, PMA, TNFalpha, prostratin) [315]. This could be explained by the very low level of Cyclin T1 in resting T cells. Li et al. also have reported that JQ1 potentiates HIV-1 reactivation by prostratin and PHA in the J-Lat A2 latent cell line [313]. In addition to BRD4, the study of Ott and Verdin has also identified BRD2 as a new Tat-independent suppressor of HIV-1 transcription in latently infected cells and underscored the therapeutic potential of BET inhibitors in the reversal of HIV latency [316].

Tat itself can be a target for therapeutic intervention at various levels [317,318]. Recently, the first crystal structure for Tat bound to P-TEFb was reported [319]. This structure resolved at 2.1 angstroms, reveals intimate contacts between Tat and the T loop of cdk9 and marked interactions of Tat and with cyclin T1. This structure could lead to the rational design of a new class of HIV-1 inhibitors.

#### The “tat code”

The interaction promiscuity of Tat cannot be explained by a single peptide. Similar to the “histone code” [320] that has been proposed to explain how histones interact with several chromatin associated factors, which can activate or repress transcription, Tat can be posttranslationally modified at various residues, thereby creating a variety of isoforms with specific functions, the “Tat code”. Phosphorylation of Tat was the first modification identified, but there has been little follow up on is role in Tat activity. Lysine acetylation has been described at Lys28 allowing high-affinity binding to P-TEFb [321,322]. Lys28 acetylation is reversed by HDAC6 [323]. Acetylation at Lys50/51 leads to the dissociation of Tat from TAR and promotes the association of the bromodomain-containing p300/CBP associated factor (PCAF) acetyltransferase [321,322,324-327]. Lys50 acetylation generates an interaction surface also for the SWI-SNF chromatin-remodeling complex [328]. Deacetylation of Tat is mediated by SIRT1 and possibly controls a late step in Tat transcriptional activity, allowing the recycling of the deacetylated protein for next rounds of transcription [329]. Lys51 is also methylated by the monomethyl-transferase Set7/9 and demethylated by the action of LSD1 [259,330]. Interestingly, both activities are required for Tat function, possibly controlling different steps of process. To this end, the dwell time calculated by fluorescence recovery after photobleaching (FRAP) of the various components present at the HIV-1 transcription site showed that Tat and Cdk9 behave similarly while RNAPII remains on the gene for a longer time required to reach the end of the gene [331,332]. These data support the idea that Tat and P-TEFb remain associated in the elongating complex, rather than Tat alone being transferred to the elongating polymerase, but that the complex dissociates from RNAPII before termination of transcription. Finally, polyubiquitination of Lys71 mediated by the proto-oncoprotein Hdm2 does not induce proteasome-dependent degradation but has an activating effect on Tat [333]. Tat interacts also with the 19S PAAF1 component of the proteasome to increase transcription in a non-proteolytic mode [334]. However, the role of Lys71 polyubiquitination in this pathway of Tat activation has not been established yet. Since posttranslational modifications are critical for Tat activity, they represent targets for antiviral therapy. For example, the structure of the Tat/PCAF interaction is being explored for the development of antiviral compounds [335-337].

#### Recruitment of the SWI /SNF chromatin-remodeling complex to the HIV-1 promoter

Integrated HIV-1 is embedded into host chromatin and shows a characteristic histone signature with the positioning of nuc-1. Remodeling of histones is required for transcription (see above). This could be achieved by specific posttranslational modification of histone tails, such as acetylation and methylation, and by remodeling complexes that require ATP to function. The SWI/SNF family complexes contain the Brahma (BRM) or homologue BRG1 ATPases together with several other components. Two distinct SWI/SNF complexes have been described: BAF and PBAF. The defining subunit of the BAF complex is BAF250a/ARID1a, and those of the PBAF complex are BAF180, BAF200, and BRD7. SWI/SNF complexes participate in the activation of HIV-1 transcription. Kashanchi and collaborators showed that Tat activates HIV-1 transcription in the G1/S phase of the cell cycle [338]. Acetylated Tat binds BRG1 and both are recruited to the 5’ LTR. BRG1 knockdown in latently infected cell lines, such as U1 and ACH2 stimulated with TNFalpha, result in a significant decrease of viral gene expression. Consistently, Verdin and collaborators have shown that knockdown of the core SWI/SNF components integrase interactor 1 (INI-1/SNF5) and BRG-1 suppresses Tat-mediated transactivation and that Tat BRG1 interaction requires Lys50/51 acetylation [328]. They also demonstrated that BRG1 and INI1 cooperate with the p300 coactivator and acetyltransferase to synergistically activate the HIV-1 LTR. The Emiliani’s laboratory demonstrated that BRM is required for proper Tat-mediated activation of the HIV-1 LTR [339]. They showed that Tat fails to transactivate the 2037 integrated LTR if BRM is depleted by siRNA in HeLa cells. Furthermore, acetylated Tat is required for the interaction with BRM. More recently, it has been reported that establishment and maintenance of HIV-1 latency requires the BAF complex, which helps positioning of the repressive nucleosome-1 immediately downstream of the HIV-1 transcriptional start site [340]. Depletion of BAF-specific subunits results in de-repression of HIV-1 latency concomitant with the loss of nuc-1. Upon transcriptional activation, BAF is lost from the HIV-1 promoter, while the PBAF complex is selectively recruited by acetylated Tat to facilitate LTR transcription. Thus, BAF and PBAF, recruited during different stages of the HIV-1 life cycle, display opposing functions on the HIV-1 promoter.

The involvement of SWI/SNF in HIV-1 is more complex since it may involve other mechanisms beyond transcription. The core SWI/SNF component INI1/SNF5 was originally identified as an interactor of HIV-1 integrase [341]. It has been proposed that by interacting with IN, INI/SNF5 interferes with early steps of HIV-1 infection [342]. More recently, it has been shown that HIV-1 integration requires nucleosome remodeling at the integration site and that this activity is mediated by the interaction of INI1/SNF5 with the viral integrase [119]. Finally, an intriguing observation by the Trono’s laboratory, which has not been investigated further, indicated that incoming HIV-1 pre-integration complexes trigger the nuclear export of INI1/SNF5 and of the nuclear body component PML (TRIM19) [343]. The HIV-1 genome appears to associate with these proteins before nuclear export. PML is sequestered in the nucleus in the presence of arsenic while the INI1/pre-integration complex interaction is disrupted and the efficiency of HIV-1 infection is increased [343]. These data suggest a repressive role for INI-1/hSNF5 in HIV-1 transcription.

### Posttranscriptional mechanisms of HIV-1 latency

Considerable attention has focused on the role of chromatin and transcription (co)factors in the control of HIV-1 latency. However, latently infected cells are also maintained in a suboptimal cellular environment for HIV-1 expression by mechanisms operating at the posttranscriptional level either by inhibiting nuclear viral RNA export or by inhibiting HIV-1 translation.

The generation of infectious retroviral progeny requires the synthesis and export to the cytoplasm of three species of RNA: (i) spliced subgenomic mRNAs for protein translation, (ii) partially spliced RNAs that function as the mRNA for the viral proteins Gag-Pol and Env and (iii) genomic unspliced full-length viral RNA. The viral protein Rev promotes the export of RNAs from the nucleus through the association to an RNA element called the Rev response element (RRE) that is present in partially spliced and genomic RNAs. Nuclear export occurs upon association of Rev with the nuclear export factor Exportin 1 (Crm-1) and translocation of the Rev/RNA complex to the cytoplasm where it is either translated or packaged into virions (for a review:[344]). Siliciano and colleagues have reported that resting CD4+ T cells from HIV-1-infected individuals on cART retain both Tat and Rev transcripts in their nuclei [345]. This finding stands in sharp contrast to the effective export of these multiply spliced viral transcripts in activated CD4+ T cells. The defect in viral RNA export could be rescued by ectopic expression of the RNA binding protein PTB (polypyrimidine tract binding protein). PTB, the PTB-associated factor PSF and Matrin 3 have all been identified as HIV-1 RNA binding factors [346-349]. Although it has been proposed that HIV-1 (partially) spliced RRE-containing HIV-1 RNAs are committed to Matrin3/Rev mediated export by PSF, their role in HIV-1 latency remains to be determined [349]. Of note, however, that Rev expression alone is able to increase HIV-1 expression in resting CD4+ T lymphocytes, indicating that defects in viral RNA export could result also from insufficient levels of Rev [350].

Critical to the study of HIV-1 latency is the choice of the cellular model. It was recently reported that the direct infection of resting CD4+ T cells by spinoculation results in the generation of a population of cells carrying integrated proviruses, that are capable of producing low levels of Gag but are unable to spread the infection [351]. The defect appeared at the level of Env production and could be reversed by T cell stimulation. This observation opens yet a novel scenario on the post-transcriptional regulation of HIV-1 latency.

**Figure 3:**
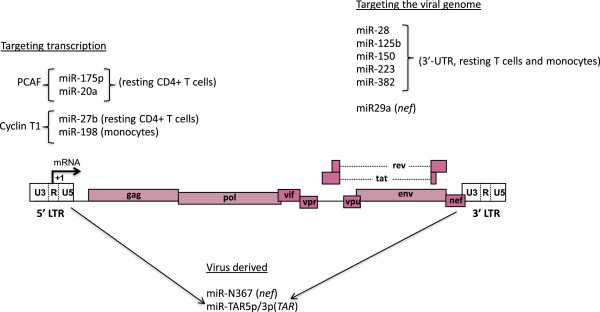
**Modulation of HIV-1 replication by miRNA.** HIV-1 impacts the cellular miRNA pathways in several ways. The figure shows a schematic representation of the HIV-1 provirus with the indicated viral genes and LTRs, including the transcription start site in the 3’LTR. PCAF and Cyclin T1, which are both essential cofactors for viral transcription, are targeted by the indicated miRNAs in resting T cells and monocytes. A cluster of miRNAs is targeted directly towards the 5'end of the HIV-1 genome and their inhibition rescues infectious virus from resting T cells. Finally two sets of miRNA that are derived from the HIV-1 genome are shown. Their location within the LTR (TAR and nef) is duplicated (arrows).

Micro RNAs (miRNA) are short non-coding single-stranded RNAs that mediate post-transcriptional gene silencing (PTGS). Following RNAPII transcription, pri-miRNA transcripts are processed in the nucleus by Microprocessor, an enzymatic complex containing Drosha. The resulting pre-miRNAs are exported to the cytoplasm and cleaved by Dicer into the mature form, which is incorporated into the RNA-induced silencing complex (RISC). The miRNA-RISC then typically binds to the 3’-untranslated region (3’UTR) of a target mRNA, leading to translational repression. Several changes in the miRNA profile of various cohorts of HIV-1 infected patients have been observed [352-354]. However, these studies are more indicative of the cellular environment of a particular patient, or cohort of patients, rather than the direct effect of viral infection given the low percentage of HIV-1 infected cells *in vivo*. Knockdown of Dicer or Drosha increases HIV-1 replication [355,356], but this phenotype could depend on complex effects on non-miRNA and miRNA pathways that influence the processing of cellular and viral miRNAs. Direct assessment of miRNA profiles changes induced by HIV-1 infection has been approached directly in linfoblastoid cell lines and/or cultured PBMCs infected *ex vivo*[352,355,357-359]. Both up-regulation and down-regulation of cellular miRNAs have been observed with different scenarios. Interestingly, Tat, and possibly Vpr, function as RNA silencing suppressors (RSS) being able to modulate miRNA expression levels in infected cells [360-362] miRNAs could inhibit HIV-1 gene expression by decreasing the levels of essential cellular co-factors and are involved in the susceptibility to HIV-1 infection of different cell types like monocytes compared to macrophages for example [363]. The first essential cofactors of viral transcription that have been shown to be modulated by miRNAs are the PCAF acetyltransferase [355] and Cyclin T1 [364-366]. PCAF is regulated by miR175p and miR20a in resting T cells, while Cyclin T1 is regulated principally by miR27b [365,366]. Cyclin T1 expression increases also during differentiation of monocytes to macrophages due the down modulation of miR198 that targets the 3’UTR [364]. In resting CD4+ T cells instead, miRNAs may also participate in repressing HIV-1 gene expression by directly targeting HIV-1 mRNAs. Five cellular miRNAs (miR-28, miR-125b, miR-150, miR-223, and miR-382) recognize the 3’-end of HIV-1 mRNAs and are upregulated in resting, but not activated, CD4+ T cells [350]. Interestingly, rescue of HIV-1 from latently infected cells could be achieved by a combination of complementary antisense miRNAs opening the way to a novel therapeutic strategy to rescue latent HIV-1. Two independent groups showed that HIV-1 *nef* gene contains a miR-29a targeted site that interferes with HIV-1 replication [356,367]. Finally, several HIV-1 derived miRNAs (called virus-derived vmiRNAs) have also been obtained by *in silico* analysis, deep sequencing of infected cells or miRNA expression profiling [358,359,368]. These include TAR-derived miRNA-TAR5p/3p [369,370] and the *nef*-derived miR-N367 [371]. TAR-derived miRNA may have an anti-apoptotic effect targeting apoptotic genes [372] or may target directly HIV-1 transcription as it has been recently proposed [289]. A summary of the principal miRNAs affecting HIV-1 is shown in Figure 3.

## Therapeutic strategies

Despite current effective and life-prolonging cART, a small population of latently infected cells persists in the human body. These HIV-1 reservoirs are insensitive to cART and able to escape from the host immune response [1,3]. These are therefore a permanent source for virus reactivation and could be responsible for the rebound of plasma viral load observed after cART interruption [1,3]. Therefore, cART treatment requires lifelong adherence, leading to several long-term side effects and a life expectancy lower than that of uninfected individuals. Moreover, several problems, such as the cost, the access for everybody, the stigma, the possibility of resistance with non-adherence, as well as the fact that intensification of cART is unlikely to lead to eradication, contribute to the necessity of finding a cure. Several therapeutic approaches aiming at achieving either a sterilizing cure (elimination of HIV-1 from the human body) or a functional cure (long-term control of HIV-1 replication and disease progression in the absence of cART) have been proposed [1,14]. Such strategies aimed at eliminating the HIV-1 reservoir have been extensively reviewed in [8,373]. In this review, we will essentially focus on strategies to induce the reactivation of HIV-1 production from latently infected cells and the purge of latent reservoirs.

### A sterilizing cure

Strategies for sterilizing cure include hematopoietic stem cell transplantation and gene therapy. The former is certainly not a feasible approach for treatment worldwide but it has provided the first evidence of virus eradication from an infected patient. The latter aims at rendering the target cells refractory to infection.

#### The “Berlin patient”

A breakthrough in HIV-1 research is the first case of a possible sterilizing cure, “the Berlin patient”. This HIV-1+ patient was treated for acute myeloid leukemia with multiple rounds of chemotherapy and hematopoietic stem cell transplantations from a donor with genetic HIV-1 resistance, a CCR5delta32 homozygous [374]. It is well established that homozygosity for a naturally occuring 32-bp deletion in the chemokine receptor CCR5, the major coreceptor for HIV-1 [375], provides resistance against HIV-1 acquisition [375-382]. The patient stopped cART the day prior to transplant and over 5 years later has not had a rebound in viremia or other indications of viral replication [383]. However, after this intervention, *in vitro* studies demonstrated that the donor-derived cells were susceptible to infection by X4 virus, but X4 variants have not emerged *in vivo*[384]. This cleverly designed strategy out of fortunate circumstances led to a patient who remained without viral rebound long after transplantation and discontinuation of antiretroviral therapy. This case has renewed hope for gene therapy in order to specifically eliminate or reduce the expression of HIV-1 coreceptor CCR5. More recently, a recent report has shown that two patients under cART with relapsed Hodgkin’s lymphoma who received a CCR5 −/+ haemopoetic stem-cell transplant, had undetectable proviral DNA and replication-competent HIV 8–17 several months after transplantation (Henrich et al. XIX International AIDS Conference, Washington DC, USA, 22–27 July 2012).

#### Gene therapy

Gene therapy has been first proposed as a treatment for HIV-1 infection in 1988 by David Baltimore with the term “intracellular immunization” [385]. Several approaches of gene therapy have been proposed in the past. Recently, RNA based strategies (ribozymes, antisense RNA, small-interfering RNA) or protein-based strategies such as zinc-finger nucleases have been implemented (reviewed in [386,387]). In brief, hematopoetic cells from patients were either transduced *ex vivo* with a retroviral vector carrying a gene for a ribozyme targeting viral RNA encoding the viral proteins Tat and Vpr [388] or transfected with a vector delivering a ribozyme targeting the CCR5 co-receptor, a short-interfering RNA targeting expression of the HIV-1 proteins Tat and Rev, and a RNA decoy to Tat [384]. The hematopoetic cells or CD4+ T cells can be also treated with a zinc-finger nuclease that is designed to specifically eliminate or reduce expression of the HIV-1 coreceptors CCR5 or CXCR4 (reviewed in [8]). The zinc-finger nuclease can be delivered by adenoviral or retroviral vectors or by nucleofection. These genetically modified cells were then transfused back into the autologous donors. Recently published clinical trials of gene therapy in HIV-1-infected patients are summarized in [8], Table 1.

### A functional cure

A functional cure aims at the long-term control of virus infection without the drawbacks of cART [389]. Structured activation of HIV-1 gene expression in latently infected cells together with an intensive antiviral regimen is proposed as an adjuvant therapy aimed at decreasing the pool of latent reservoirs. This could be combined with immune-based strategies aimed at enhancing the clearance of latently infected cells which have been induced to express HIV antigens.

#### Elite controllers and VISCONTI patients

Several examples of “natural” functional cure exist. Indeed, a group of rare HIV-1-infected patients spontaneously control HIV-1 replication in absence of cART for prolonged periods of time. These patients called “elite controllers” carry a specific HLA polymorphism and show an effective cytolytic CD8+ T-cell response and an enhanced activity of myeloid dendritic cells [390,391]. This group represents an imperfect model for a functional cure because their status is linked to a precise genetic background that cannot be reproduced therapeutically and also because they still show disease progression [390]. However, the study of genetics, viral and immune characteristics of elite controllers can teach us a lot about the criteria to be taken into account to achieve a transposable functional cure in naïve or chronic HIV-1 infected patients. In this context, several studies have shown that the reservoir of elite controllers is significantly smaller with low concentration of HIV-1 DNA in different subsets of blood cells and in tissues (reviewed in [390,391]). The reservoirs of HIV-1 establish early in the infection. Consequently, early treatment may be a potential strategy to reduce or even control the reservoir and to allow preserving immune response. In addition, rare HIV-1-infected patients acquire a controller status after prolonged cART initiated in the acute phase [392,393] or, even more rarely, in the chronic phase [394,395]. Treatment initiation during primary HIV-1 infection rather than during chronic HIV-1 infection may: i) further reduce residual viral replication [396], ii) limit viral diversity [397] and viral reservoirs [398], iii) preserve innate immunity and T/B cell functions [399-401], and iv) accelerate immune restoration [402]. Moreover, studies also show that CD4+ T cell counts are higher and that viral rebound occurs later (and at a lower level) after the discontinuation of treatment that began during primary infection compared with treatment that began during chronic HIV-1 infection [403,404]. In this context, a cohort, called VISCONTI (for Viro-Immunological Sustained Control after Treatment Interruption), is ongoing where patients have been treated with prolonged therapy that was initiated in acute HIV-1 infection and maintain both undetectable plasma viremia and low cell-associated DNA levels despite cART interruption for several years [405]. These individuals are called post-treatment controllers (PTCs). Importantly, a recent and elegant study from the same group has characterized a group of 14 PTCs [406]. These authors found that most PTCs were readily distinguishable from spontaneous HIV controllers in many respects (severe primary infection, unfavorable HLA genotypes, low CD8+ T cell activation status). They have highlighted that the control of viremia following treatment interruption was associated with very low HIV-1 blood reservoirs in the PTCs. Interestingly, five PTCs experienced a progressive decline in their viral reservoir after treatment interruption, which is one of the goals in the search for an HIV cure [406]. Akey additional element might be a low reservoir distribution in cell subsets with long lifespan as naïve and central-memory T cells. They showed that the cell subsets of all the PTCs analyzed *ex vivo* carried very low levels of HIV-1 DNA [406]. These results argue in favor of early cART initiation and open up new therapeutic perspectives for HIV-1-infected patients. However, arguments against cART initiation during primary HIV-1 infection include the potential for long-term toxicity, the development of resistant viruses and the cost. A low reservoir and a boosted immune response seem to be the two requisite criteria to allow a control of HIV-1 by the host immune system.

#### Reactivation of latent reservoirs of HIV-1

Early but unsuccessful attempts at purging the latent pool of infected cells were performed using IL-2 and other mitogenic stimuli that mimicked T cell activation (reviewed in [3]). Initial attempts to reduce the frequency of latently infected CD4+ resting T cells in patients on cART have often involved immune activation therapy approaches. These studies were based on the assumption that activation of the latent viral reservoir would result in rapid cell death due to HIV-induced cytopathic effects and that the infections virus released by these activated cells would be contained by the administration of cART. The rational for this is that eradication of latent reservoirs might be feasible through global cellular activation (by using for example IL-2, alone or in combination with an anti-CD3 monoclonal antibody (OKT3) [81,145,407]). While these therapies significantly reduced latently infected cell numbers, a rebound of plasma viremia still occurred several weeks after cART treatment interruption. Moreover, induction of generalized immune activation is not really desirable in purging strategies because it leads to severe side effects in patients and produces an abundance of activated target cells for the neo-synthesized viruses. However, this latter problem may be partially resolved by intensification of cART during the treatment period.

There is now interest in the cytokine IL-7 since it is more effective in reversing latency than IL-2 via the JAK/STAT5 signaling pathway [145]. Moreover, IL-7 has been shown to reactivate HIV-1 in primary T cells and seems well tolerated in HIV-infected patients [83,88]. The administration of IL-7 in cART-treated patients was associated with a small increase or “blip” in plasma HIV-1 RNA [408,409]. To determine whether IL-7 reduces latent HIV-1 reservoir, a clinical trial, called ERAMUNE01 directed by C. Katlama (ClinicalTrials.gov NCT01019551) is ongoing with HIV-1-infected patients receiving IL-7 in combination with an intensification of cART (Table 1). The outcomes of this trial could help in the development of new and important therapies for HIV-1 eradication, even though some concerns were reported regarding the use of IL-7. Indeed, IL-7 drives homeostatic proliferation of memory T cells and may potentially expand not only uninfected cells, but also latently infected cells by inducing proliferation of all cells, specifically transitional memory T cells [12]. IL-7 has recently been shown unable to reduce the reservoirs size in a model of HIV-1 latency [89] or to reactivate the latent viral pool in patients receiving a single dose to increase their CD4+ T cell count [410]. Therefore, the results of the ERAMUNE01 clinical trial will be of great interest.

Up-regulating cellular transcription to induce HIV-1 gene expression has been proposed as another strategy for reducing latent HIV-1 reservoirs. Recent studies have identified individual compounds that are capable of reversing HIV-1 latency without T-cell activation (described in section 6). Among those, HDACIs are the most studied in anti-HIV-1 clinical trials. In 2005, the Margolis group used VPA, a relatively weak and non-toxic HDACI, in a small pilot study in which a modest decline of the latent reservoir size was observed in three out of four patients [411]. However, latter studies have failed to demonstrate any benefit from VPA in reducing the number of latently infected resting CD4+ T cells [232,412-414]. Most recently, Routy et al. confirmed that adding VPA to stable cART does not reduce the latent HIV-1 reservoirs in virally suppressed patients [415]. This latter multicentre randomized clinical study based on 56 virologically suppressed patients has shown no significant reduction in the frequency of CD4+ T cells harboring replication-competent HIV-1 after 16 and 32 weeks of VPA therapy 500 mg twice a day [415]. A far more potent HDACI, vorinostat (SAHA), has significant potency in promoting HIV-1 replication *in vitro* from latently infected cells [224,225]. Vorinostat is licensed for the treatment of cutaneus T-cell lymphoma and relatively well tolerated in humans. Vorinostat has already entered two clinical trials to evaluate its effect on latent infection in HIV-infected patients: one directed by S. Lewin (ClinicalTrials.gov NCT01365065) at the Alfred Hospital, Melbourne, Australia and the other by D. Margolis (ClinicalTrials.gov NCT01319383) at the University of North Carolina, Chapel Hill, USA (Table 1). The first study currently evaluates the effect of vorinostat at 400 mg per day for 14 days, with a primary end point of change in cell-associated unspliced RNA in CD4+ T cells isolated from both blood and rectal tissue. Results from this study should be available in the next 1–2 years (reported in [226]). In the second study published in July 2012, the Margolis’s group reported that administration to cART-treated patients of a single clinically tolerable dose of vorinostat disrupts HIV-1 latency in blood resting CD4+ T cells. Briefly, these authors first performed validation assays by purifying the resting CD4+ T cells from 16 patients in whom viremia was fully suppressed by cART and by testing the reactivation potential of vorinostat in these *ex vivo* cultures. Following a 6 h vorinostat exposure, HIV-1 RNA expression and chromatin acetylation level were significantly up-regulated in 11 of the 16 patient cell cultures [230]. Of the 11 eligible/responder patients, 8 patients agreed to receive a single 200 mg dose of vorinostat, followed by a 400 mg dose at 4- to 5-weeks later. In all 8 patients, this exposure resulted in a significant increase in acetylation of total cellular histone H3, temporally associated with an increased (mean increase of 4.8 fold) expression of unspliced HIV-1 gag RNA within resting CD4+ T cells [230]. These results demonstrated that, at least for a period of time in some infected cells, all of the restrictions that limit the expression of latent proviral genomes have been overcome. In contrast, in this trial, the authors observed neither an alteration of residual low-level viremia nor a substantial reduction in the frequency of replication-competent HIV-1 within resting CD4+ T cells [230]. These latter results might reflect that multiple doses of vorinostat, reactivation/anti-latency combination approach and/or additional interventions are required to clear infected cells. Recently, a pilot clinical trial has started with the HDACI panobinostat (called also LBH589) directed by L. Østergaard (ClinicalTrials.gov NCT01680094) at Aarhus University Hospital, Aarhus, Denmark. The primary outcome measure is the change from baseline in HIV transcription in latently infected CD4+ T-cells as quantified by copies of unspliced HIV-RNA in the CD4+ T-cells of HIV-infected patients on suppressive cART. These issues are important goals for future studies as well as the development of HDACI with improved specificity, potency and safety profiles for the selective targeting of latent proviral genomes.

Romidepsin (called also FK288), another HDACI approved by the FDA for the treatment of cutaneous T cell lymphoma, has been shown to be one thousand fold more potent then SAHA at inducing latent HIV-1, one thousand to twenty thousand fold more potent in inhibiting HDAC enzymatic activity and induced HIV RNA expression *ex vivo* in 12 of 13 HIV + cART-treated patients (Geleziunas et al., 2012, abstract Retrovirology, 9 (Suppl1):O2). The average increase in viral RNA was approximately 8.6-fold (Geleziunas et al., 2012, abstract Retrovirology, 9 (Suppl1):O2). Therefore, the HDACI Romidepsin may be a very promising latent HIV-1 inducer *in vivo* and its favorable toxicity profiles could allow multiple-dose treatment. A clinical trial is ongoing with Romidepsin and the results are awaited with high interest. Of note, it would be very important to measure the size of the reservoirs during the course of Romidepsin administration in the HIV-1+ patients enrolled in this trial.

PKC inducers also present several advantages in the context of anti-HIV-1 purging strategies (described in section 6.2). In this context, bryostatin, the only clinically available PKC agonist [241] is currently tested with HIV-1-infected patients in a pilot clinical trial directed by S. Moreno, Department of Infectious Diseases, Ramon y Cajal Hospital, Madrid, Spain (Table 1). This ongoing trial evaluates the latent cell reservoir size, residual viremia and cell associated viral RNA after a single dose of bryostatin-1 and results are yet unavailable (Moreno S, 2012, abstract Retrovirology, 9(Suppl 1):I16).

Disulfiram (DSF), a safe and well tolerated FDA-approved drug to treat chronic alcoholism (described in section 6.2), is currently evaluated in a pilot clinical trial (ClinicalTrials.gov NCT01286259) under the direction of S. Deeks at the University of California, San Francisco, USA to assess whether a decrease in the frequency of resting CD4+ T cells harboring replication-competent HIV-1 occurs. This pilot clinical trial involved 14 patients on cART with plasma viral loads below 50 copies HIV-1 RNA/ml of plasma. DSF was administrated for two weeks and the frequency of latently infected cells as well as residual viral viremia was measured during disulfiram administration. Preliminary results from this trial revealed that disufiram administration resulted in a small transient increase in viral load in some trial participants (Spivak et al., 2012, Abstract 19th Conference on Retrovirus and Opportunistic Infections. Seattle, March 5–8). No significant change in the size of the latent reservoir was observed at these intervals.

The hypothesis was that pharmacological reactivation of latent HIV-1 would cause death of the infected cells by viral cytophatic effects or host cytolytic T lymphocytes (CTLs), thereby decreasing the size of the latent pool. Indeed, an overarching question is whether the increases in viral RNA induced either by these compounds will be sufficient to raise the amount of viral protein expression in reactivated cells to levels that lead to viral cytopathicity and/or elimination by cytotoxic T cells. In this regard, the group of Siliciano has recently reported that virus reactivation with vorinostat occurs in latently infected primary CD4+ T cells generated *in vitro*, but is not associated with death induced directly by viral protein, i.e. viral cytopathic effect [416]. Moreover, unstimulated HIV-specific CTL from cART-treated patients also fail to kill autologous latently infected CD4+ T cells after latent viruses are reactivated [416]. In contrast, antigen-specific stimulation of patient CTLs prior to virus reactivation leads to rapid killing of latently infected vorinostat-reactivated cells [416]. Moreover, monocytes-macrophages and myeloid dendritic cells are more resistant to the cytophatic effects of the virus. Indeed, macrophages have the peculiar capacity to accumulate virions in intracellular vacuolar compartments, as observed originally *in vivo* in macrophages infiltrating the brain of individuals with HIV-1 encephalopathy and AIDS-associated dementia [417]. However, macrophages could be cleared by host mechanisms if cytolytic T lymphocyte responses are boosted with HIV-1 specific antigens to more efficiently kill infected cells, in which latency has been reversed. Therefore, significant boosting of CTL responses, through therapeutic vaccination or other means, prior to virus reactivation might be an effective strategy for purging the latent pool and thereby eradicating HIV-1 infection. Several approaches of immunotherapy are reviewed in [389].

## Conclusions

Overall, cumulated data indicate that a series of complementary mechanisms are involved in mediating HIV-1 latency and reactivation. Different forms of latency probably coexist in a single patient and vary from one patient to the other. Therefore, further research in the basic mechanisms of HIV-1 latency remains a priority because pharmacological anti-latency compounds would have to be multi-pronged. Indeed, additional mechanistic studies of HIV-1 latency will identify novel targets for pharmacological approaches to reactivate latent reservoirs. Studying the epigenome and DNA methylome of the host genes following HIV-1 infection also constitutes an important issue. Such combinatory strategies to eliminate HIV-1 reservoirs with selective activators of viral expression, even if they do not achieve total eradication, could lead to a decline of HIV-1 reservoir levels sufficient to allow an efficient control of the infection by the host immune system. Lower viral loads should permit highly welcome therapeutic interruptions (“drug-free windows”). However, intentional re-ignition of HIV-1 expression by the above approaches aimed at eliminating latently infected cells has yet to be further characterized *in vivo*. The future goals in HIV-1 research are varied but inter-connected. Improvement of assays, technologies and *in vitro* (cellular) models for exploring potential reactivation strategies will be necessary. Moreover, further studies on animal models aimed at demonstrating their validity for HIV persistence should be conducted. A better characterization of latent HIV infection in non-T cell HIV reservoirs, including tissue macrophages, astrocytes and dendritic cells is also waited. An important effort remains to be done for the improvement of the anti-latency drugs. In a therapeutic goal, the ideal compounds should be orally available, active but not toxic in a wide variety of cell types (or presenting mild host toxicities if administrated for limited periods of time), unable to induce global immune activation and compatible with the different components of cART. Careful consideration of both short- and long-term toxicities, as well as activity of these compounds and quantification of their effects on viral reservoirs (including sanctuaries such as CNS and GALT) are key issues in further developing such anti-latency drugs. In addition to latency, the failure to cure HIV-1 infection is believed to be the result of T-cell dysfunction stemming from persistent immune activation. Therefore, new therapeutic strategies should also include reversal of immune exhaustion and boosting of specific anti-HIV-1 CTL immune responses.

## Abbreviations

5-Aza: 5-azacytidine; 5-Aza-CdR: 5-aza-2’-deoxycytidine; AIDS: Acquired immunodeficiency syndrome; AP-1: Activating protein-1; APOBEC3G: APOlipoprotein BmRNA editing catalytic subunit-like protein 3G; ATP: Adenosine TriPhosphate; Bcl-2: B-cell Lymphoma 2; BRD4: Bromodomain-containing protein 4; BRM: Brahma gene; cART: Combination antiretroviral therapy; CBF-1: C-promoter binding factor-1; CCR5: Chemokine CC motif receptor 5; CD4: Cluster designation 4; Cdk9: Cyclin-dependent kinase 9; c-Myc: v-myc myelocytomatosis viral oncogene homolog; CNS: Central nervous system; COUP-TF: Chicken ovalbumin upstream Promoter-transcription factor; CTD: Carboxy-terminal domain; CTIP-2: COUP-TF interacting protein 2; CTL: Cytolytic T lymphocytes; CXCR4: Chemokine CXCMotif receptor 4; CycT1: Cycline T1; DNA: Deoxyribonucleic acid; DNMT: DNA MethylTransferase; DNMTI: DNA MethylTransferase inhibitor; dNTP: DeoxyNucleotide TriPhosphate; DSF: Disulfiram; DPP: 12-DeoxyPhorbol 13-Phenylacetate; DSIF: DRB-Sensitive inducing factor; EC: Elite controllers; EZH2: Enhancer of Zeste 2; FDA: Food and Drug Administration; FIV: Feline immunodeficiency virus; FRAP: Fluorescence recovery after photobleaching; GALT: Gut-associated lymphoid tissue; GFP: Green fluorescent protein; GM-CSF: Granulocyte-macrophage colony-stimulating factor; HAT: Histone AcetylTransferase; HDAC: Histone DeACetylase; HDACI: Histone DeACetylase inhibitor; HEXIM1: HMBA inducible protein 1; HIV-1: Human immunodeficiency virus type 1; HMBA: HexaMethylene BisAcetamide; HMT: Histone MethylTransferase; HMTI: Histone MethylTransferase Inhibitor; IN: Integrase; HP1: Heterochromatin Protein 1; HPC: Hematopoietic progenitor cells; HRP-2: Hepatoma-derived growth factor related protein 2; IKB: Inhibitor of NF-kappaB; IKK: IKappaB kinase; IL-2: InterLeukin 2; Ini1: Integrase-interacting protein 1; JAK: Janus kinase; JNK: c-Jun N-terminal kinase; LARP7: La-related protein 7; LEDGF: Lens epithelial derived growth factor; LEF-1: Lymphoid enhancer factor 1; LSD1: Lysine specific demethylase 1; LSF: Late SV40 Factor; LTNP: Long-term nonprogressors; LTR: Long terminal repeat; MBD: Methyl-CpG binding domain protein; MeCP2: Methyl-CpG binding protein 2; miRNA: Micro RNA; NAP1L1: Nucleosome assembly factor 1 like 1; Nef: Negative regulatory factor; NELF: Negative eLongation factor; NFAT: Nuclear factor of activated T cells; NF-kappaB: Nuclear factor kappa B; OKT3: Orthoclone K T-cell receptor 3 antibody; PAFc: Polymerase-associated factor complex; PBMC: Peripheral blood mononuclear cell; PCAF: p300/CBP-associated factor; PIC: Preintegration complex; PKC: Protein kinase C; PMA: Phorbol 12-myristate 13-acetate; PRC2: Polycomb repressive complex 2; PSF: PTB-associated factor; PTB: Polypyrimidine tract binding protein; P-TEFb: Positive transcription elongation factor b; PTEN: Phosphatase and tensin homolog; RISC: RNA-induced silencing complex; RNAPII: RNA polymerase II; RRE: RNA element called the Rev response element; SAH: S-adenosyl-L-homocysteine; SAM: S-AdenosylMethionine; SAHA: SuberoylAnilide hydroxamic acid; SCID-hu: Severe combined immunodeficient-human mice; SEC: Super elongation complex; SET: Su(var)3-9 enhancer-of-zeste and Trithorax; SIRT: (Sirtuin) Silent mating tape information regulation 2 homolog; SIV: Simian immunodeficiency virus; Sp1: SV40-promoter specific factor; STAT: Signal transducers and activators of transcription; Suv39h1: Suppressor of variegation 3–9 homolog 1; SWI/SNF: SWItching/sucrose non fermenting; TAR: Tat responsive element; Tat: TransActivator of transcription; TCF-4: T cell factor 4; TCF-LEF: T cell factor/lymphoid enhancer factor; TCM: Central memory T cells; TCR: T-Cell receptor; TEM: Effector memory T cells; TNF-α: Tumor necrosis factor-α; TPX: Trapoxin; TRIM: Tripartite motif protein; TSA: TrichoStatin A; TTM: Transitional memory T cells; Vif: Viral infectivity factor; VPA: Valproic Acid; Vpu: Viral protein U; VSVG: Vesicular stomatitis virus G; YY1: Ying Yang protein 1; ZBRK: KRAB-zinc finger.

## Competing interests

The authors declare that they have no competing interests.

## Authors’ contributions

CVL, SB and AM contributed to the writing of the manuscript. All authors read and approved the final manuscript.
